# Approximate incidence geometry in the plane

**DOI:** 10.1007/s40687-025-00552-4

**Published:** 2025-09-05

**Authors:** Tuomas Orponen

**Affiliations:** Department of Mathematics and Statistics, University of Jyväskylä, P.O. Box 35 (MaD), 40014 Jyväskylä, Finland

## Abstract

These are lecture notes for a mini-course given in Banff in June 2024. They discuss the problem of bounding the number of $\delta$*-incidences* $\mathcal {I}_{\delta }(P,\mathcal {L}) := \{(p,\ell ) \in P \times \mathcal {L} : p \in [\ell ]_{\delta }\}$ under various hypotheses on $P \subset \mathbb {R}^{2}$ and $\mathcal {L} \subset \mathcal {A}(2,1)$. The main focus will be on hypotheses relevant for the *Furstenberg set problem*.

## Introduction

These lectures are about *approximate incidence geometry*, but they are far from a complete introduction to this topic. A more accurate name for the contents would be *introduction to upper bounds in linear approximate incidence geometry in the plane*. For further reading, I mention a few important topics **not** covered in these notes, and some of the most recent references (as of summer 2025). I will not discuss the *high-low method* pioneered in [28] and applied, e.g. in [6, 8, 9, 23, 24, 53]. I will not discuss *Falconer’s distance set problem*, or other “curvilinear” incidence problems [20, 25, 26, 36, 55, 64]. I will not discuss *incidence lower bound problems* [8, 9, 14]. Finally, I will not discuss the *Kakeya problem* [32, 59–61].

A prototypical problem in approximate incidence geometry is the following. Let $P \subset \mathbb {R}^{2}$ be a finite set, and let $\mathcal {L}$ be a finite set of lines in the plane. Fix a (small) scale parameter $\delta > 0$, and consider the $\delta$*-incidences*

$$\begin{aligned} \mathcal {I}_{\delta }(P,\mathcal {L}) := \{(p,\ell ) \in P \times \mathcal {L} : p \in [\ell ]_{\delta }\}. \end{aligned}$$

Here and below, $[A]_{\delta }$ stands for the closed Euclidean $\delta$-neighbourhood of $A \subset \mathbb {R}^{d}$.

### Question 1

(Incidences) How large can $|\mathcal {I}_{\delta }(P,\mathcal {L})|$ be in terms of *P* and $\mathcal {L}$?

The following formulation is equivalent, but often gives a useful, slightly different perspective to the problem:

### Question 2

(Rich lines) Given a finite set of points $P \subset \mathbb {R}^{2}$, and a parameter $r \geqslant 2$, how large can be the set $\mathcal {L}_{r,\delta }(P) = \{\ell \subset \mathbb {R}^{2}: |P \cap [\ell ]_{\delta }| \geqslant r\}$ of lines in $\mathbb {R}^{2}$ whose $\delta$-neighbourhood contains at least *r* points from *P*?

The answers to Questions 1–2 heavily depend on various hypotheses we place on *P* and $\mathcal {L}$. If we make no hypotheses, the best possible answer is $|\mathcal {I}_{\delta }(P,\mathcal {L})| \leqslant |P||\mathcal {L}|$. This is attained if *P* is contained in a single $(\delta /2)$-disc $B \subset \mathbb {R}^{2}$, and all the lines $\ell \in \mathcal {L}$ also intersect *B*. To avoid this situation, one typically asks—at least—that both *P* and $\mathcal {L}$ are $\delta$-separated; in the formulation of Question 2, one only counts $\delta$-separated elements in $\mathcal {L}_{r}(P)$. The $\delta$-separation of $\mathcal {L}$ is defined with respect to some natural metric on $\mathcal {A}(2,1)$, the space of all (affine) lines in $\mathbb {R}^{2}$. A common choice (also used in these notes) is

$$\begin{aligned} d_{\mathcal {A}(2,1)}(\ell _{1},\ell _{2}) := \Vert \pi _{L_{1}} - \pi _{L_{2}}\Vert + |a_{1} - a_{2}|, \end{aligned}$$

whenever $\ell _{j} = L_{j} + a_{j}$, $L_{j} \subset \mathbb {R}^{2}$ is a 1-dimensional subspace, and $a_{j} \in L_{j}^{\perp }$. The notation $\pi _{L}$ refers to orthogonal projection to *L*, and $\Vert \cdot \Vert$ is the operator norm.

If both *P* and $\mathcal {L}$ are $\delta$-separated, the sharp estimate for $|\mathcal {I}_{\delta }(P,\mathcal {L})|$ is

1.1 $$\begin{aligned} |\mathcal {I}_{\delta }(P,\mathcal {L})| \lesssim \delta ^{-1/3}|P|^{2/3}|\mathcal {L}|^{2/3}, \end{aligned}$$

see [19] (or [11, Exercise 7.5] or [21, Theorem 1.5] for alternative arguments which yield (1.1) with a $\delta ^{-\epsilon }$-loss). This bound is (e.g.) attained whenever the points form a $\delta$-net inside a $\Delta$-disc $B \subset \mathbb {R}^{2}$ for some $\delta \leqslant \Delta \leqslant 1$, and $\mathcal {L}$ a $\delta$-net of lines intersecting *B*.

Despite its sharpness, (1.1) is not very useful for solving “interesting” problems. These problems tend to involve more hypotheses on *P* and $\mathcal {L}$, which make the sharp bound for $|\mathcal {I}_{\delta }(P,\mathcal {L})|$ more difficult to prove. We will return to this in later sections.

## Bounds for $\delta$-incidences under $\sqrt{\delta }$-separation

When $\delta = 0$, we abbreviate

$$\begin{aligned} \mathcal {I}(P,\mathcal {L}) := \mathcal {I}_{0}(P,\mathcal {L}) = \{(p,\ell ) \in P \times \mathcal {L} : p \in \ell \}, \end{aligned}$$

and $\mathcal {L}_{r}(P):= \mathcal {L}_{r,0}(P)$. The “exact incidence” $p \in \ell$ makes the problem of estimating $|\mathcal {I}(P,\mathcal {L})|$ easier than the problem in Question 1, although still non-trivial. Under no additional hypotheses on *P* and $\mathcal {L}$, Szemerédi and Trotter [57] in 1983 proved that

2.1 $$\begin{aligned} |\mathcal {I}(P,\mathcal {L})| \lesssim |P|^{2/3}|\mathcal {L}|^{2/3} + |P| + |\mathcal {L}|. \end{aligned}$$

Equivalently, if $P \subset \mathbb {R}^{2}$ is finite, then

$$\begin{aligned} |\mathcal {L}_{r}(P)| \lesssim \frac{|P|^{2}}{r^{3}} + \frac{|P|}{r}, \qquad r \geqslant 2. \end{aligned}$$

The Szemerédi-Trotter bound is quite sharp. Perhaps, the simplest example is where $\mathcal {L} = \{\ell \}$, and $P \subset \ell$: then $|\mathcal {I}(P,\mathcal {L})| = |P|$. This shows that the implicit constant in front of the |*P*| (similarly $|\mathcal {L}|$) term cannot be lower than 1. This leaves open the sharpness of the term $|P|^{2/3}|\mathcal {L}|^{2/3}$. The exponent 2/3 is sharp, indeed the simple Example 2.2 below shows that the implicit constant in front of the term $|P|^{2/3}|\mathcal {L}|^{2/3}$ cannot be lower than $2^{-2/3}$:

### Example 2.2

Consider $$P:= \{1,\ldots ,n\} \times \{1,\ldots ,2n^{2}\}$$ and

$$\begin{aligned} \mathcal {L} := \{(x,ax + b) : a \in \{1,\ldots ,n\} {\text { and }} b \in \{1,\ldots ,n^{2}\}\}. \end{aligned}$$

Note that every line $\ell = \ell _{a,b} \in \mathcal {L}$ is *n*-rich, because if $a \in \{1,\ldots ,n\}$ and $b \in \{1,\ldots ,n^{2}\}$, then $ax + b \in \{1,\ldots ,2n^{2}\}$ for all $x \in \{1,\ldots ,n\}$. Thus, $|\mathcal {I}(P,\mathcal {L})| \geqslant n|\mathcal {L}| \geqslant n^{4}$. On the other hand, if (2.1) holds with constant $C > 0$, then

$$\begin{aligned} n^{4} \leqslant |\mathcal {I}(P,\mathcal {L})| \leqslant C|P|^{2/3}|\mathcal {L}|^{2/3} + C|P| + C|\mathcal {L}| = C2^{2/3}n^{4} + 2Cn^{3} + Cn^{3}. \end{aligned}$$

This can only hold for all $n \in \mathbb {N}$ if $C \geqslant 2^{-2/3} \approx 0.63$.

### Remark 2.3

The sharpest currently known form of (2.1) is $|\mathcal {I}(P,\mathcal {L})| \leqslant C|P|^{2/3}|\mathcal {L}|^{2/3} + |P| + |\mathcal {L}|$ with $C = 2.44$, see [1]. It is also known [52, Remark 4.2] that this inequality cannot hold with constant $C < 3\root 3 \of {3/(4\pi ^{2})} \approx 1.27$ (see also [3, Sect. 1.3]). In that example, $P = \{1,\ldots ,n\} \times \{1,\ldots ,n\}$, and $\mathcal {L}$ consists of $(\epsilon n)$-rich lines for any $\epsilon \in (0,1)$.

### Crossing number proof

We start with a proof of (2.1) using the *crossing number inequality* discovered by Székely [56]. This proof also yields some information about $\delta$-incidences, provided that both the points and lines are $\sqrt{\delta }$-separated:

#### Theorem 2.4

Let $\delta \in (0,1]$, and let $P \subset B(1) \subset \mathbb {R}^{2}$ be a $\sqrt{\delta }$-separated set. Let $r \geqslant 2$, and let $\mathcal {L}$ be a family of $\sqrt{\delta }$-separated lines such that $|P \cap [\ell ]_{\delta }| \geqslant r$ for all $\ell \in \mathcal {L}$. Then,

2.5 $$\begin{aligned} |\mathcal {L}| \lesssim \frac{|P|^{2}}{r^{3}} + \frac{|P|}{r}. \end{aligned}$$

Equivalently, $$|\mathcal {I}_{\delta }(P,\mathcal {L})| \lesssim |P|^{2/3}|\mathcal {L}|^{2/3} + |P| + |\mathcal {L}|$$.

Here is the crossing number inequality (proved independently in [37] and [2]):

#### Lemma 2.6

(Crossing number inequality) Let $G = (V,E)$ be a simple graph with $|E| \geqslant 4|V|$. Then, the *crossing number* $\textrm{cr}(G)$ of *G* has the lower bound

$$\begin{aligned} \textrm{cr}(G) \geqslant \frac{1}{64} \frac{|E|^{3}}{|V|^{2}}. \end{aligned}$$

The crossing number $\textrm{cr}(G)$ is the minimal number of edge crossings in any planar drawing of *G*. In particular, $\textrm{cr}(G) = 0$ if and only if *G* is a planar graph.

#### Proof of Theorem 2.4

We may assume that $\delta \leqslant \tfrac{1}{100}$, since otherwise $|P| \sim 1$, and Theorem 2.4 is trivial. Fix $\ell \in \mathcal {L}$, so $|P \cap [\ell ]_{\delta }| \geqslant r \geqslant 2$. Since the set $P \cap [\ell ]_{\delta }$ is $\sqrt{\delta }$-separated, and $\sqrt{\delta }$ is far larger than the width of the tube $[\ell ]_{\delta }$, the points in $P \cap [\ell ]_{\delta }$ lie on a $\sim \sqrt{\delta }$-Lipschitz graph over $\ell$. In particular, they have a natural ordering (say, given by their projections to $\ell$). We form a set of unordered edges $E(\ell )$, in a graph with vertex set $V = P$, by placing an edge between any pair of consecutive points *p*, *q* in this ordering. Then, we define

$$\begin{aligned} E := \bigcup _{\ell \in \mathcal {L}} E(\ell ). \end{aligned}$$

The (Euclidean) length of any edge $[p,q] \in E$ is $\geqslant \sqrt{\delta }$. It may happen that [*p*, *q*] lies in multiple families $E(\ell _{1}),\ldots ,E(\ell _{k})$, but then the associated lines $\ell _{1},\ldots ,\ell _{k}$ all lie at distance $\lesssim \sqrt{\delta }$ from each other in $\mathcal {A}(2,1)$,^1^ Since $\mathcal {L}$ was assumed $\sqrt{\delta }$-separated, it follows that $k \lesssim 1$, i.e. the families $E(\ell )$ have bounded overlap. Therefore,

$$\begin{aligned} |E| \gtrsim \sum _{\ell \in \mathcal {L}} |E(\ell )| \geqslant |\mathcal {L}|(r - 1) \gtrsim |\mathcal {L}|r, \end{aligned}$$

since $r \geqslant 2$. Now, if $|E| < 4|P|$, we have $r|\mathcal {L}| \lesssim |P|$, and therefore, $|\mathcal {L}| \lesssim |P|/r$. This bound corresponds to the second term in (2.5).

Assume then that $|E| \geqslant 4|P|$. In this case, the crossing number inequality is applicable in the graph $G = (P,E)$ and yields

$$\begin{aligned} (r|\mathcal {L}|)^{3} \lesssim \textrm{cr}(G)|P|^{2}. \end{aligned}$$

**Fig. 1:**
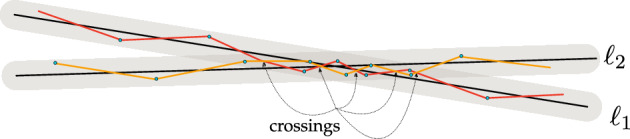
The danger of many crossings

We claim that $\textrm{cr}(G) \lesssim |\mathcal {L}|^{2}$, which will complete the proof of (2.5). The idea is roughly: every crossing pair of edges $(e_{1},e_{2})$ determines (hopefully) a unique pair of lines $(\ell _{1},\ell _{2})$ such that $e_{1} \in E(\ell _{1})$ and $e_{2} \in E(\ell _{2})$. Therefore, $\textrm{cr}(G) \lesssim |\mathcal {L}|^{2}$. The uniqueness is not entirely trivial, however. We know that the edges in $E(\ell _{j})$ lie on an $O(\sqrt{\delta })$-Lipschitz graph over $\ell _{j}$, but this alone is not helpful (unless $\delta =0$): It is well possible that two $O(\sqrt{\delta })$-Lipschitz graphs intersect each other multiple times, see Fig. 1.

What saves the day is the fact that if $\ell _{1} \ne \ell _{2}$, then all the crossings between $E(\ell _{1}),E(\ell _{2})$ must occur in a disc of radius $O(\sqrt{\delta })$. Let us do this carefully. We write

$$\begin{aligned} \textrm{cr}(G)&\leqslant \mathop {\sum _{(e_{1},e_{2})}}_{(e_{1},e_{2}) {\text { is crossing}}} |\{(\ell _{1},\ell _{2}) : e_{j} \in E(\ell _{j}), \, j \in \{1,2\}\}|\\&\leqslant \sum _{(\ell _{1},\ell _{2}) \in \mathcal {L} \times \mathcal {L}} |\{(e_{1},e_{2}) \in E : e_{j} \in E(\ell _{j}), \, j \in \{1,2\} {\text { and }} (e_{1},e_{2}) {\text { is crossing}}\}|. \end{aligned}$$

It suffices to show that individually $|\{(e_{1},e_{2}) \in E : \ldots \}| \lesssim 1$.

If $\ell _{1} = \ell = \ell _{2}$, there are no crossing pairs $(e_{1},e_{2})$ with $e_{1},e_{2} \in E(\ell )$. So, consider $\ell _{1} \ne \ell _{2}$. Let $(e_{1}^{1},e_{2}^{1}),\ldots ,(e_{1}^{m},e_{2}^{m})$ be pairs of crossing edges with $(e_{1}^{i},e_{2}^{i}) \in E(\ell _{1}) \times E(\ell _{2})$ for all $1 \leqslant i \leqslant m$. Then, every edge $e_{1}^{i}$ and $e_{2}^{i}$ meets the intersection $[\ell _{1}]_{\delta } \cap [\ell _{2}]_{\delta }$, which is contained in a disc $B \subset \mathbb {R}^{2}$ of radius $\lesssim \sqrt{\delta }$. So, in fact $e_{j}^{i} \in E(\ell _{j},B) := \{e \in E(\ell _{j}) : e \cap B \ne \varnothing \}$ for all $1 \leqslant i \leqslant m$. Thus,

$$\begin{aligned} (e_{1}^{i},e_{2}^{i}) \in E(\ell _{1},B) \times E(\ell _{2},B), \qquad 1 \leqslant i \leqslant m. \end{aligned}$$

But $|E(\ell _{j},B)| \lesssim 1$ for $j \in \{1,2\}$ by the $\sqrt{\delta }$-separation of *P*, so $m \lesssim 1$, as claimed. $\square$

### Cell decomposition proof

In this section, we give a second proof for (2.1), based on *cell decompositions*. This method was pioneered by Szemerédi and Trotter, but a simpler implementation can be achieved by the use of polynomials (see Lemma 2.11). The cell decomposition proof of (2.1) could also be modified to yield the $\sqrt{\delta }$-separated version stated in Theorem 2.4. We leave this as a (reasonably challenging) exercise for the reader.

We repeat below exactly what we are planning to prove:

#### Theorem 2.7

Let $P \subset \mathbb {R}^{2}$ be a finite set. Then,

2.8 $$\begin{aligned} |\mathcal {L}_{r}(P)| \lesssim \frac{|P|^{2}}{r^{3}} + \frac{|P|}{r}, \qquad r \geqslant 2. \end{aligned}$$

Besides the idea of cell decompositions, a second ingredient is the following simple incidence estimate:

#### Proposition 2.9

For finite $P \subset \mathbb {R}^{2}$ and $\mathcal {L} \subset \mathcal {A}(2,1)$,

2.10 $$\begin{aligned} |\mathcal {I}(P,\mathcal {L})| \lesssim |P|^{1/2}|\mathcal {L}| + |P|. \end{aligned}$$

#### Proof

For $p \in P$, write $\mathcal {L}(p) := \{\ell \in \mathcal {L} : p \in \ell \}$. Using the definition of $\mathcal {I}(P,\mathcal {L})$ and Cauchy–Schwarz:

$$\begin{aligned} |\mathcal {I}(P,\mathcal {L})| = \sum _{p \in P} |\mathcal {L}(p)| \leqslant |P|^{1/2} \Big ( \sum _{p} |\mathcal {L}(p)|^{2} \Big )^{1/2} = |P|^{1/2} \Big ( \sum _{\ell ,\ell '} |P \cap \ell \cap \ell '| \Big )^{1/2}. \end{aligned}$$

Splitting the sum into diagonal and off-diagonal parts (where $\ell = \ell '$ and $\ell \ne \ell '$, respectively), we obtain $|\mathcal {I}(P,\mathcal {L})| \leqslant |P|^{1/2} (|\mathcal {I}(P,\mathcal {L})| + |\mathcal {L}|^{2})^{1/2}$. This yields (2.10). $\square$

Here is the lemma from [27] on polynomial cell decompositions, we will need:

#### Lemma 2.11

Let $d \in \mathbb {N}$ be a degree. If $P \subset \mathbb {R}^{n}$ is a finite set, there exists a polynomial surface $Z = \{\textrm{poly} = 0\}$ with $\textrm{poly} \ne 0$ of degree $\deg Z := \deg (\textrm{poly}) = d$ such that every component $O \subset \mathbb {R}^{d} {\setminus } Z$ contains $\lesssim |P|/d^{n}$ points of *P*.

#### Remark 2.12

The components $O \subset \mathbb {R}^{2} {\setminus } Z$ will be the “cells” in the “cell decomposition” proof of Theorem 2.7. Szemerédi and Trotter did not have access to Lemma 2.11, so they crafted their “cells” using straight lines and line segments. In many interesting, non-trivial cases (for example when *P* is a product set to begin with) crafting the cells by hand (using vertical and horizontal line) is easy.

#### Remark 2.13

The cases $2 \leqslant r \lesssim 1$ of Theorem 2.7 are easy and can be obtained from the following 2*-ends argument*. Let $\mathcal {L}$ be a set of lines such that $|P \cap \ell | \geqslant r \geqslant 2$ for all $\ell \in \mathcal {L}$. In particular, every $\ell \in \mathcal {L}$ contains $\gtrsim r^{2}$ pairs of **distinct** points $(p,q) \in (P \cap \ell )^{2}$. As $\ell \in \mathcal {L}$ varies, these pairs are distinct, so we obtain

$$\begin{aligned} r^{2}|\mathcal {L}| \leqslant \sum _{\ell \in \mathcal {L}} |(P \cap \ell )^{2}| \leqslant |P|^{2}. \end{aligned}$$

Rearranging yields $|P| \gtrsim r|\mathcal {L}|^{1/2}$. For $2 \leqslant r \sim 1$, this coincides with the bound from Theorem 2.7. A key idea underlying the cell decomposition proof of Theorem 2.7 is to decompose *P* into smaller pieces $P \cap O$, where we “expect” $2 \leqslant |P \cap O \cap \ell | \lesssim 1$ (although this inequality does not literally appear in the proof).

We are then equipped to prove Theorem 2.7.

#### Proof

We may assume that $r \geqslant 4$, since the opposite case follows from Remark 2.13. Write $\mathcal {L}:= \mathcal {L}_{r}(P)$. We may also assume that $|\mathcal {L}| \geqslant r$. Namely when $|\mathcal {L}| \leqslant r$, we may use the elementary bound (2.10) to deduce that

$$\begin{aligned} r|\mathcal {L}| \leqslant |\mathcal {I}(P,\mathcal {L})| \lesssim |P|^{1/2}|\mathcal {L}| + |P|. \end{aligned}$$

If the second term dominates, then $|\mathcal {L}| \lesssim |P|/r$, and if the first term dominates, then $|P|^{2} \gtrsim r^{4}$, and therefore $|\mathcal {L}| \leqslant r \lesssim |P|^{2}/r^{3}$.

Assume $|\mathcal {L}| \geqslant r$, and apply Lemma 2.11 with degree $d:= \lfloor (r/2)\rfloor$ to obtain a non-trivial polynomial surface $Z \subset \mathbb {R}^{2}$ with $\deg (Z) \leqslant d$ such that $|P \cap O| \lesssim |P|/r^{2}$ for all connected components $O \subset \mathbb {R}^{2} {\setminus } Z$. These components will be denoted $\mathcal {O}$.

Let

$$\begin{aligned} \mathcal {L}_{Z} := \{\ell \in \mathcal {L} : \ell \subset Z\} \quad {\text {and}} \quad \mathcal {L}_{O} := \mathcal {L} {\setminus } \mathcal {L}_{Z}. \end{aligned}$$

Since *Z* can contain $\leqslant d = \lfloor r/2\rfloor$ distinct lines,^2^ We infer that $|\mathcal {L}_{Z}| \leqslant \lfloor r/2\rfloor$, and therefore $|\mathcal {L}_{Z}| \leqslant \tfrac{1}{2}|\mathcal {L}|$, using the assumption $|\mathcal {L}| \geqslant r$. Thus $|\mathcal {L}| \leqslant 2|\mathcal {L}_{O}|$, and it suffices to verify the bound (2.8) for $\mathcal {L}_{O}$ in place of $\mathcal {L}$.

Fix $\ell \in \mathcal {L}_{O}$. By assumption $|\ell \cap P| \geqslant r \geqslant 4$. In particular, $\ell$ contains $\geqslant r - 1 \geqslant 3r/4$ *segments* of the form [*x*, *y*] with $x,y \in P$ distinct and consecutive (i.e. there are no points of *P* between *x* and *y*). Let $\mathcal {I}$ be the collection of all such segments. A segment $I = [x,y] \in \mathcal {I}$ is *cellular* if $x,y \in O$ for a common component $O \subset \mathbb {R}^{2} {\setminus } Z$, otherwise *I* is *non-cellular*. Note that every line $\ell \in \mathcal {L}_{O}$ contains $\leqslant d = \lfloor r/2\rfloor$ non-cellular segments. Indeed, $\ell$ intersects *Z* somewhere on each non-cellular segment. So, if the number of non-cellular segments were $> d$, then $|Z \cap \ell | > d$, and hence $\ell \subset Z$ (contrary to the definition of $\mathcal {L}_{O}$).

The cellular segments are denoted $\mathcal {I}_{O}$, and their number is

$$\begin{aligned} |\mathcal {I}_{\mathcal {O}}| = \sum _{O \in \mathcal {O}} |\{I \in \mathcal {I}_{O} : I \subset O\}| \leqslant \sum _{O \in \mathcal {O}} |P \cap O|^{2} \lesssim \frac{|P|}{r^{2}} \sum _{O \in \mathcal {O}} |P \cap O| \leqslant \frac{|P|^{2}}{r^{2}}. \end{aligned}$$

Every line $\ell \in \mathcal {L}_{O}$ contains $\geqslant 3r/4$ segments, but only $\leqslant \lfloor r/2\rfloor$ non-cellular segments. Therefore, $\ell$ contains $\geqslant r/4$ cellular segments, denoted $\mathcal {I}_{O}(\ell )$. When $\ell \in \mathcal {L}_{O}$ varies, the collections $\mathcal {I}_{O}(\ell )$ are clearly disjoint. Therefore,

$$\begin{aligned} r|\mathcal {L}| \lesssim \tfrac{r}{4} \cdot |\mathcal {L}_{O}| \leqslant \sum _{\ell \in \mathcal {L}_{O}} |\mathcal {I}_{O}(\ell )| \leqslant |\mathcal {I}_{O}| \lesssim |P|^{2}/r^{2}. \end{aligned}$$

Consequently $|P| \gtrsim r^{3/2}|\mathcal {L}|$, as claimed. $\square$

## Bounds for $\delta$-incidences under *s*-dimensional separation

In Theorem 2.4, we saw that the Szemerédi-Trotter bound for $\delta$-incidences is valid under the hypothesis that both *P* and $\mathcal {L}$ are $\sqrt{\delta }$-separated. For applications in “continuum” incidence problems in fractal geometry, this hypothesis on $\mathcal {L},P$ is not so natural, see Remark 3.3. To keep the discussion concrete, we now introduce one distinguished “continuum” incidence question, which will follow us for the rest of these notes.

### Problem 1

(*Furstenberg set problem*) Let $s \in (0,1]$ and $t \in [0,2]$. Let $F \subset \mathbb {R}^{2}$ be an (*s*, *t*)*-Furstenberg set*. This means that there exists a *t*-dimensional family $\varnothing \ne \mathcal {L} \subset \mathcal {A}(2,1)$ such that $\dim _{\textrm{H}}(F \cap \ell ) \geqslant s$ for all $\ell \in \mathcal {L}$. What is the best lower bound for $\dim _{\textrm{H}}F$?

The Furstenberg set problem (for $t = 1$) was proposed by Wolff [62, 63] in the late 90 s. After plenty of partial progress [4, 13, 15, 22, 28–30, 34, 38, 48, 49], it was finally solved in 2023 by Ren and Wang [53]. The sharp lower bound for $\dim _{\textrm{H}}F$ is

3.1 $$\begin{aligned} \dim _{\textrm{H}}F \geqslant \mathfrak {f}(s,t) := \min \{s + t,(3s + t)/2,s + 1\}. \end{aligned}$$

Here is a simple but illustrative example of Furstenberg sets:

### Example 3.2

For $s \in [0,1]$, let $F = C \times \mathbb {R}$, where *C* is an *s*-dimensional Cantor set. Then $\dim _{\textrm{H}}F = s + 1$, and *F* is an (*s*, 2)-Furstenberg set: Every non-vertical line $\ell \subset \mathbb {R}^{2}$ satisfies $\dim _{\textrm{H}}(F \cap \ell ) = \dim _{\textrm{H}}C = s.$ In particular, the term “$s + 1$” in (3.1) cannot be omitted.

### Remark 3.3

The estimate (3.1) can be viewed as a continuum analogue of the Szemerédi-Trotter bound (2.1). We clarify this somewhat informally. Instead of aiming for (3.1), let us attempt to prove (the box dimension estimate) $|F|_{\delta } \geqslant \delta ^{-\mathfrak {f}(s,t)}$ for all $\delta > 0$ small enough. Here $|\cdot |_{\delta }$ is the $\delta$-covering number.

Pick a $\delta$-separated subset $\mathcal {L}_{\delta } \subset \mathcal {L}$ with $|\mathcal {L}_{\delta }| \approx \delta ^{-t}$, and for each $\ell \in \mathcal {L}_{\delta }$ a $\delta$-separated set $P_{\delta }(\ell ) \subset F \cap \ell$ with $|P_{\delta }(\ell )| \approx \delta ^{-s}$. Let $P_{\delta }$ be a maximal $\delta$-separated set in $\bigcup _{\ell \in \mathcal {L}_{\delta }} P_{\delta }(\ell ) \subset F$. Then,

3.4 $$\begin{aligned} |\mathcal {I}_{\delta }(P_{\delta },\mathcal {L}_{\delta })| \gtrapprox |\mathcal {L}_{\delta }|\delta ^{-s} \approx \delta ^{-(s + t)}. \end{aligned}$$

(We used here that every line in $\mathcal {L}_{\delta }$ is $\delta$-incident to $\approx \delta ^{-s}$ points in $P_{\delta }$, although these points may not be the ones in $P_{\delta }(\ell )$.) On the other hand, **if** the Szemerédi-Trotter bound was valid under the $\delta$-separation hypotheses on $P_{\delta }$ and $\mathcal {L}_{\delta }$, we could estimate

$$\begin{aligned} |\mathcal {I}_{\delta }(P_{\delta },\mathcal {L}_{\delta })| \lesssim |P_{\delta }|^{2/3}|\mathcal {L}_{\delta }|^{2/3} + |P_{\delta }| + |\mathcal {L}_{\delta }| \approx |P_{\delta }|^{2/3}\delta ^{-2t/3} + |P_{\delta }| + \delta ^{-t}. \end{aligned}$$

Combining these estimates would lead to $|F|_{\delta } \geqslant |P_{\delta }| \gtrapprox \min \{\delta ^{-(s + t)},\delta ^{-(3\,s + t)/2}\}$, and eventually $\overline{\dim }_{\textrm{B}}F \geqslant \min \{s + t,(3\,s + t)/2\}$.

First, this is too good to be true: the term “$s + 1$” in (3.1) is necessary, as we saw in Example 3.2. Second, the Szemerédi-Trotter bound is not valid under $\delta$-separation alone; the sharp bound under this hypothesis was mentioned in (1.1). The argument above using (1.1) would produce the unsharp estimate $\overline{\dim }_{\textrm{B}}F \geqslant (3s + t - 1)/2$.

One might try to use Theorem 2.4, namely the version of Szemerédi-Trotter valid under $\delta ^{1/2}$-separation. This approach can—at best—yield an unsharp estimate. The argument above also runs into a difficulty: after extracting suitable $\delta ^{1/2}$-separated sets $\mathcal {L}_{\delta ^{1/2}}$ and $P_{\delta ^{1/2}}$, it seems hard to maintain any lower bound on the $\delta$-incidences $\mathcal {I}_{\delta }(P_{\delta ^{1/2}},\mathcal {L}_{\delta ^{1/2}})$.

### Discretising incidence problems involving Hausdorff dimension

We have seen that sharp upper bounds on $|\mathcal {I}_{\delta }(P,\mathcal {L})|$ under $\delta$-separation or $\sqrt{\delta }$-separation hypotheses on *P* and $\mathcal {L}$ fail to produce sharp estimates in the (*s*, *t*)-Furstenberg set problem. This is not surprising: The hypotheses $\dim _{\textrm{H}}\mathcal {L} \geqslant t$ and $\dim _{\textrm{H}}(F \cap \ell ) \geqslant s$ involve Hausdorff dimension, and that information is lost when passing to (merely) $\delta$-separated or $\sqrt{\delta }$-separated subsets. We need to consider a separation condition that keeps the “Hausdorff dimension information”. Such a condition was introduced by Katz and Tao [34] in 2000:

#### Definition 3.5

($(\delta ,s)$-*set*) Let (*X*, *d*) be a metric space, and $C,\delta ,s > 0$. A $\delta$-separated set $P \subset X$ is called a $(\delta ,s,C)$*-set* if

$$\begin{aligned} |P \cap B(x,r)| \leqslant C\left( \frac{r}{\delta } \right) ^{s}, \qquad x \in X, \, r \geqslant \delta . \end{aligned}$$

If the value of the constant $C > 0$ is irrelevant, a $(\delta ,s,C)$-set may be called a $(\delta ,s)$-set.

We also extend the definition of $(\delta ,s)$-sets to families of dyadic cubes. For $\delta \in 2^{-\mathbb {N}}$, a family of dyadic cubes $\mathcal {P} \subset \mathcal {D}_{\delta }$ is a $(\delta ,s,C)$-set if

$$\begin{aligned} |\mathcal {P} \cap Q| \leqslant C\left( \frac{r}{\delta } \right) ^{s}, \qquad Q \in \mathcal {D}_{r}, \, r \geqslant \delta , \end{aligned}$$

where $$\mathcal {P} \cap Q = \{p \in \mathcal {P}: p \subset Q\}$$.

#### Remark 3.6

The $(\delta ,s)$-sets of these notes are sometimes called *Katz-Tao* $(\delta ,s)$*-sets* in the literature, while “$(\delta ,s)$-set” refers to sets $P \subset X$ satisfying $|P \cap B(x,r)|_{\delta } \lesssim r^{s}|P|$.

#### Example 3.7

The property of being a $(\delta ,s)$-set gets weaker as *s* increases. In fact, every finite $\delta$-separated set in $\mathbb {R}^{d}$ is a $(\delta ,d)$-set.

#### Example 3.8

Every $\sqrt{\delta }$-separated set $P \subset B(1) \subset \mathbb {R}^{2}$ is a $(\delta ,1)$-set, because $|P \cap B(x,r)| \leqslant 1$ for $\delta \leqslant r \leqslant \sqrt{\delta }$. On the other hand, for $\sqrt{\delta } \leqslant r \leqslant 1$, any *r*-disc can contain at most $\lesssim (r/\sqrt{\delta })^{2} \leqslant r/\delta$ discs of radius $\sqrt{\delta }$. In particular $|P \cap B(x,r)| \lesssim r/\delta$ for $\sqrt{\delta } \leqslant r \leqslant 1$.

**Fig. 2:**
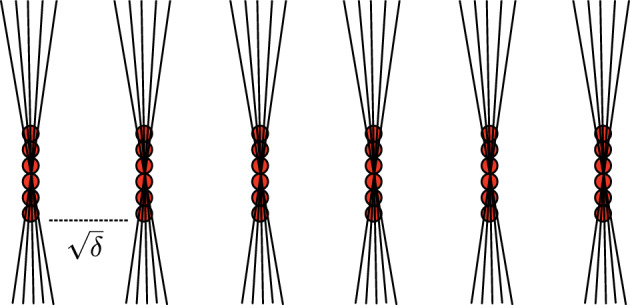
The train tracks example

Jump ahead to Fig. 2 for a rather “opposite” example of a $(\delta ,1)$-set.

We now record two standard tools for discretising continuum incidence problems involving Hausdorff dimension. Proposition 3.9 allows to find large $(\delta ,s)$-subsets, while Proposition 3.12 allows to find covers by $(\delta ,s)$-sets. We first recall that, for $s > 0$, the *s-dimensional Hausdorff content* of $K \subset \mathbb {R}^{d}$ is defined by

$$\begin{aligned} \mathcal {H}^{s}_{\infty }(K) := \inf \Big \{ \sum _{j \in \mathbb {N}} \operatorname {diam}(E_{j})^{s}\Big \}, \end{aligned}$$

where the $\inf$ runs over all countable set families $\{E_{j}\}_{j \in \mathbb {N}}$ such that $K \subset \bigcup _{j \in \mathbb {N}} E_{j}$.

#### Proposition 3.9

(Large $(\delta ,s)$-subsets) Let $K \subset \mathbb {R}^{d}$ be a bounded set with $\mathcal {H}^{s}_{\infty }(K) =: \tau > 0$. Then, for every $\delta \in (0,1]$, there exists a $(\delta ,s,C_{d})$-set $P \subset K$, and $|P| \gtrsim _{d} \tau \delta ^{-s}$.

The proof is taken from [18, Proposition A.1]. The idea is the same as in the proof of Frostman’s lemma (see, e.g. [40, Theorem 8.8]).

#### Proof

Without loss of generality, assume that $\delta = 2^{-k}$ for some $k \in \mathbb {N}$ (if $\delta \in (0,1]$ is arbitrary, first prove the proposition for the largest element in $2^{-\mathbb {N}} \cap (0,\delta ]$). We may also assume that *K* is contained in some dyadic cube $Q_{0} \in \mathcal {D}_{k_{0}}$ with $k_{0} \leqslant k$.

Let $P_{0}$ be a finite set containing exactly one point from $K \cap Q$ for all $Q \in \mathcal {D}_{2^{-k}}(K)$. Then, $|P_{0}| = |K|_{\delta } \gtrsim \tau \delta ^{-s}$ as desired, but $P_{0}$ may fail the $(\delta ,s)$-set condition. We modify $P_{0}$ as follows. Fix $Q^{k - 1} \cap \mathcal {D}_{2^{k - 1}}(P_{0})$. If

$$\begin{aligned} |P_{0} \cap Q^{k - 1}| > \left( \tfrac{\ell (Q^{k - 1})}{\delta }\right) ^{s}, \end{aligned}$$

define $P_{1} \cap Q^{k - 1} \subset P_{0} \cap Q^{k - 1}$ to be a maximal set with $|P_{1} \cap Q^{k - 1}| \leqslant (\ell (Q^{k - 1})/\delta )^{s}$. Then automatically

$$\begin{aligned} \tfrac{1}{2}\left( \tfrac{\ell (Q^{k - 1})}{\delta }\right) ^{s} \leqslant |P_{1} \cap Q^{k - 1}| \leqslant \left( \tfrac{\ell (Q^{k - 1})}{\delta }\right) ^{s}. \end{aligned}$$

Repeat this for all cubes $Q^{k - 1} \in \mathcal {D}_{2^{k - 1}}$ to obtain $P_{1}$. Then, repeat the procedure at all dyadic scales between $\delta = 2^{-k}$ and $2^{-k_{0}}$: if $P_{j}$ has already been defined, and there is a cube $Q^{k - (j + 1)} \in \mathcal {D}_{2^{k - (j + 1)}}$ such that

$$\begin{aligned} |P_{j} \cap Q^{k - (j + 1)}| > \left( \tfrac{\ell (Q^{k - (j + 1)})}{\delta }\right) ^{s}, \end{aligned}$$

choose a subset $P_{j + 1} \cap Q^{k - (j + 1)} \subset P_{j} \cap Q^{k - (j + 1)}$ satisfying

3.10 $$\begin{aligned} \tfrac{1}{2}\left( \tfrac{\ell (Q^{k - (j + 1)})}{\delta }\right) ^{s} \leqslant |P_{j + 1} \cap Q^{k - (j + 1)}| \leqslant \left( \tfrac{\ell (Q^{k - (j + 1)})}{\delta }\right) ^{s}. \end{aligned}$$

The process stops in finitely many steps, because $P_{0} \subset Q_{0}$.

We claim that for every $x \in P_{0}$ (and not only $x \in P$!) there exists a dyadic cube $Q_{x} \subset Q_{0}$ such that $\ell (Q_{x}) \geqslant \delta$ and

3.11 $$\begin{aligned} |P \cap Q_{x}| \geqslant \tfrac{1}{2}\left( \tfrac{\ell (Q_{x})}{\delta }\right) ^{s}. \end{aligned}$$

If $x \in P$, then (3.11) holds for $Q_{x} \in \mathcal {D}_{2^{-k}}(\{x\})$. If $x \in P_{0} {\setminus } P$, the point *x* was deleted from $P_{0}$ at some stage. Let $Q_{x}$ be the largest cube containing *x*, where point deletion occurred. If this happened while defining $P_{j + 1}$, then (3.10) holds with $Q_{k - (j + 1)} = Q_{x}$. But since $Q_{x}$ was the largest cube containing *x* where point deletion occurred, $P_{j + 1} \cap Q_{x} = P \cap Q_{x}$. This gives (3.11).

Let $\mathcal {Q}$ be the maximal elements in $\{Q_{x} : x \in P_{0}\}$. Then, $\mathcal {Q}$ is a disjoint cover of *K*, because every element of $\mathcal {D}_{\delta }(P_{0}) = \mathcal {D}_{\delta }(K)$ is contained in some element of $\mathcal {Q}$, as we just showed. This combined with (3.11) yields

$$\begin{aligned} |P| = \sum _{Q \in \mathcal {Q}} |P \cap Q| \gtrsim \delta ^{-s}\sum _{Q \in \mathcal {Q}} \ell (Q)^{s} \gtrsim _{d} \kappa \delta ^{-s}. \end{aligned}$$

We claim that *P* is a $(\delta ,s,C_{d})$-set. For $Q \in \mathcal {D}_{l}$ with $k_{0} \leqslant l \leqslant k$, (3.10) yields $|P \cap Q| \leqslant (\ell (Q)/\delta )^{s}$. This implies $|P \cap B(x,r)| \lesssim _{d} (r/\delta )^{s}$ for all $x \in \mathbb {R}^{2}$ and $r \geqslant \delta$. $\square$

#### Proposition 3.12

(Covering by $(\delta ,s)$-sets) Let $0 < s \leqslant d$, and let $K \subset \mathbb {R}^{d}$ be a compact set with $\mathcal {H}^{s}(K) = 0$. Then, for every $k_{0} \in \mathbb {N}$, there exists a sequence of $(2^{-k},s,1)$-sets $\{\mathcal {P}_{k}\}_{k \geqslant k_{0}}$ such that $\mathcal {P}_{k} \subset \mathcal {D}_{2^{-k}}$, and

$$\begin{aligned} K \subset \bigcup _{k \geqslant k_{0}} \cup \mathcal {P}_{k}. \end{aligned}$$

A slightly different version of this proposition was first proved by Katz and Tao in [34], but for more recent incarnations, see [23, 45].

#### Proof of Proposition 3.12

Let $K \subset \mathbb {R}^{d}$ be a compact set with $\mathcal {H}^{s}(K) = 0$. Consider the following dyadic variant of the Hausdorff content:

$$\begin{aligned} \mathcal {H}^{s}_{\delta ,\infty }(K) := \min _{\mathcal {Q}} \Big \{ \sum _{Q \in \mathcal {Q}} \ell (Q)^{s} : K \subset \bigcup _{Q \in \mathcal {Q}} Q \Big \}, \end{aligned}$$

where the “$\min$” only runs over families $\mathcal {Q}$ of dyadic cubes with side-length $\geqslant \delta$. It will be crucial that the “$\min$” exists thanks to the restriction to “large” cubes.

Fix $k_{0} \in \mathbb {N}$. Using the compactness of *K*, and $\mathcal {H}^{s}_{\infty }(K) = 0$, one may find $\delta > 0$ such that $\mathcal {H}^{s}_{\delta ,\infty }(K) \leqslant 2^{-k_{0}s}$.^3^ This means that there exists a (finite) cover of *K* by dyadic cubes $\mathcal {Q}$ such that $\ell (Q) \geqslant \delta$ for all $Q \in \mathcal {Q}$, and

3.13 $$\begin{aligned} \sum _{Q \in \mathcal {Q}} \ell (Q)^{s} = \mathcal {H}^{s}_{\delta ,\infty }(K) \leqslant 2^{-k_{0}s}. \end{aligned}$$

It turns out that the sub-families $\mathcal {P}_{k} := \{Q \in \mathcal {Q} : \ell (Q) = 2^{-k}\}$, $k \in \mathbb {N}$, are automatically $(2^{-k},s,1)$-sets, that is,

$$\begin{aligned} |\{Q \in \mathcal {P}_{k} : Q \subset \textbf{Q}\}| \leqslant \left( \tfrac{r}{2^{-k}} \right) ^{s}, \qquad \textbf{Q} \in \mathcal {D}_{r}, \, r \geqslant 2^{-k}. \end{aligned}$$

If this inequality failed for some $\textbf{Q} \in \mathcal {D}_{r}$, a new competitor for $\mathcal {H}_{\delta ,\infty }^{s}(K)$ is obtained by

$$\begin{aligned} \mathcal {Q}' := (\mathcal {Q} \cup \{\textbf{Q}\}) {\setminus } \{Q \in \mathcal {P}_{k} : Q \subset \textbf{Q}\}. \end{aligned}$$

Moreover, the analogue of the sum (3.13) for $\mathcal {Q}'$ is strictly smaller than the one for $\mathcal {Q}$, because

$$\begin{aligned} \ell (\textbf{Q})^{s} = r^{s} < |\{Q \in \mathcal {P}_{k} : Q \subset \textbf{Q}\}| \cdot 2^{-ks} = \sum _{Q \subset \textbf{Q}} \ell (Q)^{s}. \end{aligned}$$

This violates the minimality of the sum (3.13).

To summarise, we have now found a cover of *K* by $(2^{-k},s,1)$-sets, and it follows from (3.13) that $k \geqslant k_{0}$ for all (non-empty) families $\mathcal {P}_{k}$. $\square$

### Incidences between $(\delta ,s)$-sets of points and lines

We return to the problem of estimating $|\mathcal {I}_{\delta }(P,\mathcal {L})|$. After the introduction of $(\delta ,s)$-sets, a natural question is the following:

#### Problem 2

Let $0 \leqslant \alpha ,\beta \leqslant 2$. Assume that $P \subset B(1) \subset \mathbb {R}^{2}$ is a $(\delta ,\alpha )$-set, and $\mathcal {L} \subset \mathcal {A}(2,1)$ is a $(\delta ,\beta )$-set. How large can $|\mathcal {I}_{\delta }(P,\mathcal {L})|$ be in terms of

- $$\alpha $$ and $$\beta $$?
- |*P*| and $$|\mathcal {L}|$$?

We use here the notation “$\alpha$” and “$\beta$” to avoid confusion with the parameters “*s*” and “*t*” appearing in the Furstenberg set problem.

A sharp answer to part (a) was found by Fu and Ren in [22], for all values of $\alpha ,\beta \in [0,2]$. They provide an explicit continuous function $f :[0,2]^{2} \rightarrow [0,\infty )$ such that

3.14 $$\begin{aligned} |\mathcal {I}_{\delta }(P,\mathcal {L})| \lesssim _{\epsilon } \delta ^{-f(\alpha ,\beta ) - \epsilon }, \qquad \epsilon > 0, \end{aligned}$$

whenever $P,\mathcal {L}$ are as in Problem 2. In particular $f(\alpha ,\beta ) \leqslant (1 + \alpha + \beta )/2$ for $\alpha + \beta \leqslant 3$. They also give examples showing that $f(\alpha ,\beta )$ is the minimal function satisfying (3.14). The full formula for the (piecewise linear) function *f* is a bit complicated, see [22, Theorem 1.4]. The method of [22] also gives an answer to part (b), but I’m not sure if that is sharp for all cardinalities |*P*| and $|\mathcal {L}|$. We will prove the bound $f(\alpha ,\beta ) \leqslant (1 + \alpha + \beta )/2$ in Theorem 3.22. For now, let us compare Problem 2 with the Furstenberg set problem.

First some bad news: Even a sharp answer to Problem 2 does **not** solve all the cases of the (*s*, *t*)-Furstenberg set problem. The reason is, roughly, that the hypothesis $\dim _{\textrm{H}}(F \cap \ell ) \geqslant s$ available for every (*s*, *t*)-Furstenberg set is very strong, and the extremal pairs $(P,\mathcal {L})$ for Problem 2.7 do not satisfy (a $\delta$-discretised version) of this lower bound, e.g. in the form that $P \cap [\ell ]_{\delta }$, $\ell \in \mathcal {L}$, would contain large $(\delta ,\sigma )$-sets for some $\sigma > 0$.

Then, some good news: A sharp answer to Problem 2.7 still solves the cases $t \in [2 - s,2]$ of the Furstenberg set problem. In fact, we have the following proposition:

#### Proposition 3.15

Assume that $f :[0,2]^{2} \rightarrow [0,\infty )$ is a continuous function with the following property. If $P \subset B(1)$ is a $(\delta ,\alpha )$-set and $\mathcal {L} \subset \mathcal {A}(2,1)$ is a $(\delta ,\beta )$-set, then

$$\begin{aligned} |\mathcal {I}_{\delta }(P,\mathcal {L})| \leqslant \delta ^{-f(\alpha ,\beta )} \end{aligned}$$

for all $\delta > 0$ small enough. Then, every compact (*s*, *t*)-Furstenberg set $F \subset \mathbb {R}^{2}$ satisfies

3.16 $$\begin{aligned} f(\dim _{\textrm{H}}F,t) \geqslant s + t. \end{aligned}$$

#### Remark 3.17

The compactness hypothesis in Proposition 3.15 could be relaxed to boundedness, but verify this, we would need a version of Proposition 3.12 for bounded sets. This is true, see [23, Lemma 2].

By (3.14), the explicit Fu-Ren function *f* satisfies the hypothesis (3.16) up to the harmless “$\epsilon$”, so one can derive lower bounds for $\dim _{\textrm{H}}F$ from the inequality (3.16).

The bound $f(\alpha ,\beta ) \leqslant (1 + \alpha + \beta )/2$ for $\alpha + \beta \leqslant 3$, proven in Theorem 3.22, leads to the lower bound $\dim _{\textrm{H}}F \geqslant s + 1$ for (*s*, *t*)-Furstenberg sets with with $s + t \geqslant 2$. Here’s why. Assume that $s + t = 2$ (without loss of generality), and assume that there exists an (*s*, *t*)-Furstenberg set $F \subset \mathbb {R}^{2}$ with $\dim _{\textrm{H}}F < s + 1$. Then $\dim _{\textrm{H}}F + t \leqslant 3$, so

$$\begin{aligned} 2 = s + t {\mathop {\leqslant }\limits ^{(3.16)}} f(\dim _{\textrm{H}}F,t) \leqslant (1 + \dim _{\textrm{H}}F + t)/2, \end{aligned}$$

and this can be rearranged to $\dim _{\textrm{H}}F \geqslant 3 - t = s + 1$. This is the sharp bound (predicted by (3.1)) in the range $s + t \geqslant 2$. The reader is encouraged to check that a similar application of Proposition 3.15 in the range $s + t < 2$ would only yield unsharp results.

#### Proof of Proposition 3.15

By definition, there exists a line set $\mathcal {L} \subset \mathcal {A}(2,1)$ with $\dim _{\textrm{H}}\mathcal {L} \geqslant t$ such that $\dim _{\textrm{H}}(F \cap \ell ) \geqslant s$ for all $\ell \in \mathcal {L}$. It is easy to reduce matters to the case where our hypotheses are slightly strengthened as follows:

- $$\mathcal {H}^{t}_{\infty }(\mathcal {L}) \geqslant 1$$.
- $$\mathcal {H}_{\infty }^{s}(F \cap \ell ) \geqslant 1$$ for all $$\ell \in \mathcal {L}$$.

Fix $\alpha > \dim _{\textrm{H}}F$. According to the Katz-Tao covering lemma, Proposition 3.12, we may find a sequence $\{\mathcal {P}_{k}\}_{k \geqslant k_{0}}$ of families of dyadic cubes such that $\mathcal {P}_{k} \subset \mathcal {D}_{2^{-k}}$ is a $(2^{-k},\alpha )$-set, and

$$\begin{aligned} F \subset \bigcup _{k \geqslant k_{0}} \cup \mathcal {P}_{k}. \end{aligned}$$

In particular, for $\ell \in \mathcal {L}$ fixed, the intersection $F \cap \ell$ is covered by the sets $\cup \mathcal {P}_{k}$ for every $\ell \in \mathcal {L}$. By our assumption (b), and the sub-additivity of Hausdorff content, there exists an index $k(\ell ) \geqslant k_{0}$ such that $\mathcal {H}^{s}_{\infty }(\cup \mathcal {P}_{k} \cap \ell ) \gtrsim k(\ell )^{-2}$.

For $k \geqslant k_{0}$, let $\mathcal {L}_{k} := \{\ell \in \mathcal {L} : k(\ell ) = k\}$. By assumption (a), there exists $k \geqslant k_{0}$ such that $\mathcal {H}^{t}_{\infty }(\mathcal {L}_{k}) \gtrsim k^{-2}$. We now set $\delta := 2^{-k}$ for this index $k \geqslant k_{0}$, and we abbreviate $\mathcal {P} := \mathcal {P}_{k}$. Then, $\mathcal {P}$ is a $(\delta ,\alpha )$-set. Define $P \subset \cup \mathcal {P}$ by selecting one point in each square in $\mathcal {P}$.

Since $\mathcal {H}^{t}_{\infty }(\mathcal {L}_{k}) \gtrsim k^{-2} = (\log 1/\delta )^{-2}$, by the subset finding lemma, Proposition 3.9, there exists a $(\delta ,t)$-set $\bar{\mathcal {L}} \subset \mathcal {L}_{k}$ of cardinality $|\bar{\mathcal {L}}| \gtrapprox \delta ^{-t}$. Here, the “$\approx$” notation hides factors of order $\log (1/\delta )$. Moreover, since $k(\ell ) = k$ for all $\ell \in \bar{\mathcal {L}}$, we know that

3.18 $$\begin{aligned} \mathcal {H}^{s}_{\infty }(\cup \mathcal {P} \cap \ell ) \gtrapprox 1, \qquad \ell \in \bar{\mathcal {L}}. \end{aligned}$$

This implies that $|P \cap [\ell ]_{2\delta }| \gtrapprox \delta ^{-s}$ for $\ell \in \bar{\mathcal {L}}$, and consequently,

3.19 $$\begin{aligned} |\mathcal {I}_{2\delta }(P,\bar{\mathcal {L}})| \gtrapprox |\bar{\mathcal {L}}|\delta ^{-s} \gtrapprox \delta ^{-(s + t)}. \end{aligned}$$

On the other hand, since *P* is a $(\delta ,\alpha )$-set and $\bar{\mathcal {L}}$ is a $(\delta ,t)$-set, we have by the definition of the function *f*:

$$\begin{aligned} |\mathcal {I}_{2\delta }(P,\bar{\mathcal {L}})| \lesssim \delta ^{-f(\alpha ,t)}. \end{aligned}$$

These inequalities are only compatible (for all small $\delta > 0$) if $f(\alpha ,t) \geqslant s + t$. Since $\alpha > \dim _{\textrm{H}}F$ was arbitrary, we infer $f(\dim _{\textrm{H}}F,t) \geqslant s + t$ by the continuity of *f*. $\square$

### The train tracks example

We will shortly prove the bound $f(\alpha ,\beta ) \leqslant (1 + \alpha + \beta )/2$ in Problem 2, in particular $f(1,1) \leqslant 3/2$. Let us first see why this is sharp.

#### Example 3.20

Consider the points *P* and lines $\mathcal {L}$ drawn in the *train tracks example* in Fig. 2. There are $|P| = \delta ^{-1}$ points organised in $1/\sqrt{\delta }$ “columns”. Both the separation and height of the columns are $\sqrt{\delta }$. There are also $|\mathcal {L}| \sim \delta ^{-1}$ lines, with $\sim 1/\sqrt{\delta }$ lines incident to each column. In fact, every point $p \in P$ is contained in $[\ell ]_{\delta }$ for $\sim 1/\sqrt{\delta }$ lines $\ell \in \mathcal {L}$. Therefore,

$$\begin{aligned} |\mathcal {I}_{\delta }(P,\mathcal {L})| = \sum _{p \in P} |\{\ell \in \mathcal {L} : p \in [\ell ]_{\delta }\}| \sim \sqrt{\delta }^{-1}|P| = \delta ^{-3/2}. \end{aligned}$$

Both *P* and $\mathcal {L}$ are $(\delta ,1)$-sets. Therefore, under the hypothesis that both *P* and $\mathcal {L}$ are $(\delta ,1)$-sets, the sharp upper bound for $|\mathcal {I}_{\delta }(P,\mathcal {L})|$ cannot beat $\delta ^{-3/2}$. This is weaker than the Szemerédi-Trotter bound in Theorem 2.4, which would predict $|\mathcal {I}_{\delta }(P,\mathcal {L})| \lesssim (|P||\mathcal {L}|)^{2/3} + |P| + |\mathcal {L}| \leqslant \delta ^{-4/3}$.

#### Remark 3.21

Recall from Remark 3.8 that $\sqrt{\delta }$-separated sets are $(\delta ,1)$-sets. The train tracks example demonstrates that the $\sqrt{\delta }$-separation of *P* and $\mathcal {L}$ in Theorem 2.4 cannot be relaxed to the hypothesis that both *P* and $\mathcal {L}$ are $(\delta ,1)$-sets.

One can next ask (at least) the following two questions:

(i) Would it suffice to assume that only one of the sets *P* or $\mathcal {L}$ is $\sqrt{\delta }$-separated—say *P* for concreteness—while $\mathcal {L}$ is merely $\delta$-separated, or perhaps a $(\delta ,1)$-set?
(ii) Are the train tracks the only sharpness example if both *P*, $\mathcal {L}$ are $(\delta ,1)$-sets?

The answer to (i) is negative, as far as I know, but turns positive if one additionally assumes that |*P*| is “maximal”, that is $|P| \sim \delta ^{-1}$. Guth, Solomon, and Wang [28, Theorem 1.1] proved that if $P \subset B(1)$ is $\sqrt{\delta }$-separated with $|P| \sim \delta ^{-1}$, and $\mathcal {L} \subset \mathcal {L}_{r,\delta }(P)$ is $\delta$-separated, then

$$\begin{aligned} |\mathcal {L}| \lesssim \delta ^{-\epsilon } \frac{|P|^{2}}{r^{3}}, \qquad r \geqslant \delta ^{-\epsilon }, \, \epsilon > 0. \end{aligned}$$

In fact, this is only a special case of their result. The article [28] pioneered the powerful *high-low method* for studying $\delta$-incidences. We will not cover the high-low method in these notes, but the only reason is the lack of space and time. The high-low method is a central tool in $\delta$-discretised incidence geometry and was also used in the resolution of the (*s*, *t*)-Furstenberg set conjecture.

Regarding question (ii), the answer is roughly positive, except that the individual “tracks” (the columns of red balls in Fig. 2) may be rotated and translated arbitrarily, as long as the $(\delta ,1)$-set conditions are preserved. The precise statement is that if $(P,\mathcal {L})$ is a pair of $(\delta ,1)$-sets with $|\mathcal {I}_{\delta }(P,\mathcal {L})| \approx \delta ^{-3/2}$, then $P \times \mathcal {L}$ contains $\approx \delta ^{-1/2}$ “cliques” $P' \times \mathcal {L}'$ such that $|\mathcal {I}_{\delta }(P',\mathcal {L}')| \approx |P'||\mathcal {L}'| \approx \delta ^{-1}$. This is a recent result of the author and Yi [51]. In Fig. 2, the “cliques” are the pairs $P' \times \mathcal {L}'$ corresponding to each “track”.

### Cases $t \in [2 - s,2]$ of the Furstenberg set problem

In this section, we prove a special case of Fu and Ren’s theorem [22, Theorem 1.4], which yields $f(\alpha ,\beta ) \leqslant (1 + \alpha + \beta )/2$ (in the notation of (3.14)), for $\alpha + \beta \leqslant 3$. As discussed in Remark 3.17, this solves the (*s*, *t*)-Furstenberg set problem for $t \in [2 - s,2]$.

#### Theorem 3.22

Let $s + t < 3$, and $A,B \geqslant 1$. Let $P \subset B(1) \subset \mathbb {R}^{2}$ be a $(\delta ,s,A)$-set, and let $\mathcal {L} \subset \mathcal {A}(2,1)$ be a $(\delta ,t,B)$-set. Then,

3.23 $$\begin{aligned} |\mathcal {I}_{\delta }(P,\mathcal {L})| \lesssim _{s,t} \sqrt{AB\delta ^{-1}|P||\mathcal {L}|}. \end{aligned}$$

#### Remark 3.24

The bound (3.23) with an additional $\delta ^{-\epsilon }$-factor follows from [22, Theorem 1.5] and was originally proved using the high-low method. A finite field precedent is due to Vinh [58]. The $\delta ^{-\epsilon }$-free proof below is from [46], and it is based on classical Sobolev smoothing properties of the *X*-ray transform. Let us note that the idea of using *X*-ray transforms and related operators to study incidences has been applied several times prior to [46], for example in [17, 33].

We then gather some preliminaries for the proof of Theorem 3.22. The arguments are a little sketchy, for full details see [46].

#### Definition 3.25

(*X*-*ray transform*) For $f \in \mathcal {S}(\mathbb {R}^{2})$, we define the *X-ray transform* $Xf \in C(\mathcal {A}(2,1))$ by the formula

$$\begin{aligned} (Xf)(\ell ) := \int _{\ell } f \, \textrm{d}\mathcal {H}^{1}. \end{aligned}$$

The *X*-ray transform is $L^{2}$-smoothing of order $\tfrac{1}{2}$, see [42, Theorem 5.3]. The following (special case) is [46, Theorem 2.16]:

#### Proposition 3.26

For every $\chi \in C_{c}^{\infty }(\mathbb {R}^{2})$, there exists a constant $C_{\chi } > 0$ such that

$$\begin{aligned} \Vert X(f\chi )\Vert _{\dot{H}^{s + 1/2}} \leqslant C_{\chi }\Vert f\Vert _{\dot{H}^{s}}, \qquad f \in \mathcal {S}(\mathbb {R}^{2}), \, -\tfrac{1}{2} \leqslant s \leqslant \tfrac{1}{2}. \end{aligned}$$

The norm on the right hand side is the standard homogeneous Sobolev norm

$$\begin{aligned} \Vert f\Vert _{\dot{H}^{s}}^{2} := \int |\hat{f}(\xi )|^{2}|\xi |^{2\,s} \, \textrm{d}\xi . \end{aligned}$$

On the left hand side, the Sobolev norm $\dot{H}^{s + 1/2}$ is defined for functions in $\mathcal {A}(2,1)$: This can be accomplished rigorously by identifying $\mathcal {A}(2,1)$ with $[0,1] \times \mathbb {R}$, and then defining the Sobolev norm on $[0,1] \times \mathbb {R}$ instead of $\mathcal {A}(2,1)$. We do not need the details here, except for the inequality

3.27 $$\begin{aligned} \int fg \leqslant \Vert f\Vert _{\dot{H}^{r}}\Vert g\Vert _{\dot{H}^{-r}}. \end{aligned}$$

The proof of this inequality can be summarised as Plancherel + Cauchy–Schwarz.

Sobolev norms of negative order can be expressed in terms of the Riesz energy as follows: If $\mu$ is a Radon measure on $\mathbb {R}^{d}$, then

3.28 $$\begin{aligned} \Vert \mu \Vert _{\dot{H}^{(s - d)/2}}^{2} = \int |\hat{\mu }(\xi )|^{2}|\xi |^{s - d} \, \textrm{d}\xi \sim _{d,s} \iint \frac{\textrm{d}\mu (x) \, \textrm{d}\mu (y)}{|x - y|^{s}} =: I_{s}(\mu ), \qquad s \in (0,d). \end{aligned}$$

For a proof, see [40, Lemma 12.12]. Regarding Riesz energies, we need the following elementary computation concerning measures supported on $(\delta ,s)$-sets:

#### Lemma 3.29

Let $P \subset B(1) \subset \mathbb {R}^{d}$ be a $(\delta ,s,C)$-set with $s \in (0,d)$ and $C \geqslant 1$. Consider the measure $\mu := \delta ^{s}\mathcal {H}^{0}|_{P}$, and let $\mu _{\delta }:= \mu *\varphi _{\delta }$ be a standard mollification of $\mu$. Then,

$$\begin{aligned} I_{\sigma }(\mu _{\delta }) := \iint \frac{\textrm{d}\mu _{\delta }(x) \, \textrm{d}\mu _{\delta }(y)}{|x - y|^{\sigma }} \lesssim _{\sigma } C|P|\delta ^{2\,s - \sigma }, \qquad \sigma \in (s,d). \end{aligned}$$

#### Proof sketch

The mollification causes small technicalities omitted here. Essentially the proof boils down to the computation

$$\begin{aligned} \delta ^{2\,s}\sum _{p \ne q} \frac{1}{|p - q|^{\sigma }} \lesssim \delta ^{2\,s} \sum _{p \in P} \sum _{\delta \leqslant r \leqslant 1} r^{-\sigma }|P \cap B(p,r)| \leqslant A\delta ^{s}|P| \sum _{\delta \leqslant r \leqslant 1} r^{s - \sigma } \sim _{\sigma } A|P|\delta ^{2\,s - \sigma }, \end{aligned}$$

using $$s - \sigma < 0$$. $$\square $$

We are then ready to prove Theorem 3.22.

#### Proof of Theorem 3.22

Recall that $s + t < 3$. Choose $\sigma > s$ and $\tau > t$ such that $\sigma + \tau = 3$. Note that either $\sigma > 1$ or $\tau > 1$, and we assume with no loss of generality that $\tau > 1$.

Let $P \subset B(1)$ be a $(\delta ,s,A)$-set, and let $\mathcal {L} \subset \mathcal {A}(2,1)$ be a $(\delta ,t,B)$-set. We associate the following measures $\mu$ and $\nu$ to *P* and $\mathcal {L}$, respectively:

$$\begin{aligned} \mu := \delta ^{s}\mathcal {H}^{0}|_{P} \quad {\text {and}} \quad \nu := \delta ^{t}\mathcal {H}^{0}|_{\mathcal {L}}. \end{aligned}$$

One may now check that

$$\begin{aligned} |\mathcal {I}_{\delta }(P,\mathcal {L})| \lesssim \delta ^{-(s + t)} \iint \textbf{1}_{[\ell ]_{\delta }}(p) \, \textrm{d}\mu (p) \, \textrm{d}\nu _{\delta }(\ell ). \end{aligned}$$

Here $\nu _{\delta } = \nu *\varphi _{\delta }$ is a mollification of $\nu$ (to make sense of this, identify $\mathcal {A}(2,1)$ with $[0,1] \times \mathbb {R}$). The inner integral is closely related to the *X*-ray transform of $\mu$ evaluated at $\ell$: in fact

$$\begin{aligned} \int \textbf{1}_{[\ell ]_{\delta }}(p) \, \textrm{d}\mu (p) \lesssim \delta \cdot (X\mu _{\delta })(\ell ), \qquad \ell \in \mathcal {A}(2,1), \end{aligned}$$

where $\mu _{\delta } = \mu *\varphi _{\delta }$ is a mollification of $\mu$. Therefore,

3.30 $$\begin{aligned} |\mathcal {I}_{\delta }(P,\mathcal {L})| \lesssim \delta ^{1 - (s + t)}\int (X\mu _{\delta }) \, \textrm{d}\nu _{\delta }. \end{aligned}$$

Next, we use the “duality” inequality (3.27) with $r = \tau /2 - 1$, and eventually the boundedness of *X* between homogeneous Sobolev spaces (Proposition 3.26):

$$\begin{aligned} \int (X\mu _{\delta }) \, \textrm{d}\nu _{\delta } \lesssim \Vert X\mu _{\delta }\Vert _{\dot{H}^{1 - \tau /2}}\Vert \nu _{\delta }\Vert _{\dot{H}^{\tau /2 - 1}} \lesssim \Vert \mu _{\delta }\Vert _{\dot{H}^{(1 - \tau )/2}}\Vert \nu _{\delta }\Vert _{\dot{H}^{\tau /2 - 1}}. \end{aligned}$$

Since $\tau \in (1,2)$ and $\sigma + \tau = 3$, we may use Lemma 3.29 to deduce

$$\begin{aligned} \Vert \mu _{\delta }\Vert _{\dot{H}^{(1 - \tau )/2}}^{2} {\mathop {\sim _{\tau }}\limits ^{(3.28)}} I_{3 - \tau }(\mu _{\delta }) = I_{\sigma }(\mu _{\delta }) \lesssim _{\sigma } A|P|\delta ^{2\,s - \sigma }, \end{aligned}$$

and similarly $\Vert \nu _{\delta }\Vert _{\dot{H}^{\tau /2 - 1}}^{2} \sim I_{\tau }(\nu _{\delta }) \lesssim B|\mathcal {L}|\delta ^{2t - \tau }$. Plugging these estimates back into (3.30),

$$\begin{aligned} |\mathcal {I}_{\delta }(P,\mathcal {L})| \lesssim \delta ^{1 - (s + t)}\sqrt{A|P|\delta ^{2\,s - \sigma }B|\mathcal {L}|\delta ^{2t - \tau }} = \sqrt{AB\delta ^{2 - \sigma - \tau }|P||\mathcal {L}|} = \sqrt{AB\delta ^{-1}|P||\mathcal {L}|}, \end{aligned}$$

recalling that $$\sigma + \tau = 3$$. $$\square $$

### Cases $t \in [0,s]$ of the Furstenberg set problem

Every (*s*, *t*)-Furstenberg set with $0 \leqslant t \leqslant s$ satisfies $\dim _{\textrm{H}}F \geqslant s + t$. This follows from [38] or [31, Theorem A.1] (the special case $s = t$ was contained in [30]). In contrast with the cases $t \in [2 - s,2]$, the sharp result in any of the cases $t \in [0,2 - s)$ cannot be derived by combining Proposition 3.15 with Fu and Ren’s (sharp) bound for $|\mathcal {I}_{\delta }(P,\mathcal {L})|$ for $(\delta ,s)$-sets *P* and $(\delta ,t)$-sets $\mathcal {L}$.

#### Exercise 1

What is the best lower bound you can obtain on the Hausdorff dimension of (*s*, *t*)-Furstenberg sets via Proposition 3.15, when $t \in [0,2 - s)$?

#### Remark 3.31

It may appear puzzling that Fu and Ren’s sharp upper bound on $\delta$-incidences between does not always yield a sharp lower bound on the dimension of (*s*, *t*)-Furstenberg sets. To illustrate the reason, fix $\alpha ,\beta \in [0,2]$, $s \in [0,1]$, and consider the following two questions concerning a $(\delta ,\alpha )$-set $P \subset B(1)$ and a $(\delta ,\beta )$-set or lines $\mathcal {L} \subset \mathcal {A}(2,1)$:

1. Is it possible that $|P \cap [\ell ]_{\delta }| \geqslant \delta ^{-s}$ for all $\ell \in \mathcal {L}$?
2. Is it possible that $P \cap [\ell ]_{\delta }$ contains a $(\delta ,s)$-set with cardinality $\sim \delta ^{-s}$ for all $\ell \in \mathcal {L}$?

The Fu-Ren bound always gives the correct answer to (Q1), but fails to see the difference between (Q1) and (Q2)—because the non-concentration conditions on $P,\mathcal {L}$, and the number of $\delta$-incidences are the same in both questions. So, whenever $(\alpha ,\beta ,s)$ is a triple relevant for the (*s*, *t*)-Furstenberg set problem such that (Q1)-(Q2) have opposite answers, the Fu-Ren bound fails to yield a sharp estimate on Furstenberg sets.

For example, take $\alpha = 1 = \beta$ and $s = \tfrac{1}{2}$. Now the answer to (Q1) is positive thanks to the “train tracks” Example 3.20. The answer to (Q2) is negative: A positive answer would essentially show that *P* is a $(\tfrac{1}{2},1)$-Furstenberg set with “dimension” 1. However, it has been known since the early 2000 s [4, 34] that this is not possible. Indeed, now we know that $(\tfrac{1}{2},1)$-Furstenberg sets have dimension $\geqslant \tfrac{4}{3}$ by (3.1).

Remark 3.31 gives hint for how to proceed dealing with the cases $t \in [0,2 - s)$: We need variants of incidence bounds which take into account the extra information present in (Q2). Consider the following generalised notion of $\delta$-incidences:

#### Definition 3.32

Let $P \subset \mathbb {R}^{2}$, and let $\mathcal {F}$ be an arbitrary collection of subsets of $\mathbb {R}^{2}$. We define $\mathcal {I}_{\delta }(P,\mathcal {F}):= \{(p,F) \in P \times \mathcal {F}: p \in [F]_{\delta }\}$.

Our familiar $\mathcal {I}_{\delta }(P,\mathcal {L})$ is a special case of this definition. We will apply the definition in a case where each $F \in \mathcal {F}$ is a $(\delta ,s)$-subset of a line. In this context have the following incidence bound, which can be viewed as a $\delta$-incidence counterpart of Proposition 2.9:

#### Proposition 3.33

Let $P \subset B(1)$ be $\delta$-separated. Let $0 \leqslant t \leqslant s \leqslant 1$. Let $\mathcal {F} = \{F(\ell ) : \ell \in \mathcal {L}\}$ be a family of sets, where $\mathcal {L}$ is a $(\delta ,s,A)$-set, and each $F(\ell ) \subset \ell$ is individually a $(\delta ,s,B)$-set. Then,

$$\begin{aligned} |\mathcal {I}_{\delta }(P,\mathcal {F})| \lessapprox \sqrt{AB\delta ^{-s}|P||\mathcal {F}|} + |P|. \end{aligned}$$

#### Remark 3.34

This proposition is “folklore” in the sense that the proof strategy has appeared in many places; I’m not sure where first, but it is certainly implicit, e.g. in the proof of [31, Theorem A.1]. A more explicit appearance is [48, Proposition 2.13].

#### Proof of Proposition 3.33

Write

$$\begin{aligned} |\mathcal {I}_{\delta }(P,\mathcal {F})|&= \sum _{p \in P} |\{\ell \in \mathcal {L} : p \in [F(\ell )]_{\delta }\}|\\&\leqslant |P|^{1/2} \Big ( \sum _{p \in P} |\{(\ell ,\ell ') : p \in [F(\ell )]_{\delta } \cap [F(\ell ')]_{\delta }\}| \Big )^{1/2}\\&= |P|^{1/2} \Big ( \sum _{\ell ,\ell '} |P \cap [F(\ell )]_{\delta } \cap [F(\ell ')]_{\delta }| \Big )^{1/2}. \end{aligned}$$

The value of the “diagonal” sum is precisely $|\mathcal {I}_{\delta }(P,\mathcal {F})|$. If the “diagonal” sum dominates, we obtain after rearranging $|\mathcal {I}_{\delta }(P,\mathcal {F})| \lesssim |P|$.

For the “off-diagonal” sum with $\ell \ne \ell '$, observe that $[F(\ell )]_{\delta } \cap [F(\ell ')]_{\delta } \subset [\ell ]_{\delta } \cap [\ell ']_{\delta }$ is contained in a ball $B_{\ell ,\ell '} \subset \mathbb {R}^{2}$ of radius $\lesssim \delta /d_{\mathcal {A}(2,1)}(\ell ,\ell ')$. Using the $\delta$-separation of *P*, and the $(\delta ,s,B)$-set property of the sets $F(\ell )$, we deduce

$$\begin{aligned} |P \cap [F(\ell )]_{\delta } \cap [F(\ell ')]_{\delta }| \lesssim |F(\ell ) \cap B_{\ell ,\ell '}| \lesssim Bd_{\mathcal {A}(2,1)}(\ell ,\ell ')^{-s}. \end{aligned}$$

Therefore, using the $(\delta ,s,A)$-set property of $\mathcal {L}$ (which is implied by the $(\delta ,t,A)$-set property since $t \leqslant s$),

$$\begin{aligned} \sum _{\ell \ne \ell '} |P \cap [F(\ell )]_{\delta } \cap [F(\ell ')]_{\delta }| \lesssim A\sum _{\ell \ne \ell '} \frac{1}{d_{\mathcal {A}(2,1)}(\ell ,\ell ')^{s}} \lessapprox AB\delta ^{-s}|\mathcal {L}|. \end{aligned}$$

Since $|\mathcal {L}| = |\mathcal {F}|$, this completes the proof. $\square$

#### Corollary 3.35

Every (*s*, *t*)-Furstenberg set $F \subset \mathbb {R}^{2}$ with $0 \leqslant t \leqslant s$ satisfies $\dim _{\textrm{H}}F \geqslant s + t$.

#### Remark 3.36

Corollary 3.35 is evidently sharp: for example any product set $F = A \times B$ with $\dim _{\textrm{H}}A = s$ and $\dim _{\textrm{H}}B = t$ is an (*s*, *t*)-Furstenberg set, and it is often (if not always) the case that $\dim _{\textrm{H}}(A \times B) = s + t$.

#### Proof of Corollary 3.35

The proof is similar to the proof of Proposition 3.15, we only point out the differences. We make a counter assumption: $\dim _{\textrm{H}}F < \sigma + t$ for some $\sigma < s$.

Extract the $(\delta ,\sigma + t)$-set $\mathcal {P} \subset \mathcal {D}_{\delta }$ and the $(\delta ,t)$-set $\bar{\mathcal {L}} \subset \mathcal {L}$ exactly the same way as in the proof of Proposition 3.15. Recall from (3.18) that

$$\begin{aligned} \mathcal {H}^{s}_{\infty }(\cup \mathcal {P} \cap \ell ) \gtrapprox 1, \qquad \ell \in \bar{\mathcal {L}}. \end{aligned}$$

In the proof of Proposition 3.15, this information was used in the (wasteful) way to deduce that $|P \cap [\ell ]_{2\delta }| \gtrapprox \delta ^{-s}$. We now do something more sophisticated: applying the subset finding lemma, Proposition 3.9, we locate, for each $\ell \in \bar{\mathcal {L}}$, a $(\delta ,s)$-set $F(\ell ) \subset \ell$ such that $|P \cap [F(\ell )]_{2\delta }| \gtrapprox \delta ^{-s}$. Writing $\mathcal {F} := \{F(\ell ) : \ell \in \bar{\mathcal {L}}\}$, we then have

$$\begin{aligned} |\mathcal {I}_{2\delta }(P,\mathcal {F})| \gtrapprox \delta ^{-s}|\bar{\mathcal {L}}| \gtrapprox \delta ^{-s - t}. \end{aligned}$$

Compare this lower bound with (3.19)!

The family $\mathcal {F}$ satisfies the hypotheses of Proposition 3.33, so

$$\begin{aligned} \delta ^{-s - t} \lessapprox |\mathcal {I}_{2\delta }(P,\mathcal {F})| \lessapprox \sqrt{\delta ^{-s}|P||\bar{\mathcal {L}}|} + |P| \end{aligned}$$

Recalling that $|P| \lesssim \delta ^{-\sigma - t}$ with $\sigma < s$, and $|\bar{\mathcal {L}}| \lesssim \delta ^{-t}$, we arrive at a contradiction. It is worth noting that the $(\delta ,\sigma + t)$-property of *P* was not used here in any other ways than to ensure that *P* is $\delta$-separated, and $|P| \lesssim \delta ^{-\sigma - t}$ (this actually means that the Katz–Tao covering lemma, Proposition 3.12, is not really needed in the proof). $\square$

### Formal $\delta$-discretisation of the Furstenberg set problem

We discussed above (Remark 3.31) that the full solution of the (*s*, *t*)-Furstenberg set problem cannot be obtained from Problem 2 (incidences between $(\delta ,\alpha )$-sets of points and $(\delta ,\beta )$-sets of lines). There nonetheless exists a $\delta$-discretised incidence problem which implies lower bounds for the (*s*, *t*)-Furstenberg set problem. This is formalised by the next proposition:

#### Proposition 3.37

Fix $s \in [0,1]$, $t \in [0,2]$, and $\mathfrak {f} \in [0,2]$. Consider the following statement (P):

(P) For every $\eta > 0$, there exists $\epsilon > 0$ such that the following holds. Let $\mathcal {L} \subset \mathcal {A}(2,1)$ be a $(\delta ,t,\delta ^{-\epsilon })$-set with $|\mathcal {L}| \geqslant \delta ^{-t + \epsilon }$. For every $\ell \in \mathcal {L}$, let $F(\ell ) \subset B(1) \cap \ell$ be a $(\delta ,s,\delta ^{-\epsilon })$-set with $|F(\ell )| \geqslant \delta ^{-s + \epsilon }$. Then, the union $F:= \bigcup _{\ell \in \mathcal {L}} F(\ell )$ satisfies
  $$\begin{aligned} |F|_{\delta } \geqslant \delta ^{-\mathfrak {f} + \eta }. \end{aligned}$$

If (P) holds for some $s,t,\mathfrak {f}$, then every (*s*, *t*)-Furstenberg set $F \subset \mathbb {R}^{2}$ satisfies $\dim _{\textrm{H}}F \geqslant \mathfrak {f}$.

In fact, Ren and Wang [53] showed precisely that (P) in the previous proposition holds with the choice $\mathfrak {f} = \mathfrak {f}(s,t) = \min \{s + t,(3\,s + t)/2,s + 1\}$ as in (3.1).

Proposition 3.37 is proven by a pigeonholing argument similar to the ones we have seen in Proposition 3.15 and Corollary 3.35, see [31, Lemma 3.3] for the details.

### Cases (*s*, 1) under maximal separation

We have previously covered the cases $t \in [0,s] \cup [2 - s,2]$ of the (*s*, *t*)-Furstenberg set problem. The remaining cases $t \in (s,2 - s)$ are more complicated, and beyond the scope of these notes.

In this section, we establish property (P) in Proposition 3.15 with $\mathfrak {f} = (3s + 1)/2$ (the sharp value) for all pairs (*s*, 1) with $s \in (0,1]$, but under the additional hypothesis that the $(\delta ,s)$-sets $F(\ell ) \subset \ell$ are maximally separated. This is a special case of [21, Theorem 4], but the proof we present here is different, and avoids using the crossing number lemma.

For technical convenience, we work under the hypothesis that $F(\ell )$ contains a $\delta ^{s}$-net in $B(1) \cap \ell$. A modification of the argument would, however, work under the hypotheses that $|F(\ell )| \approx \delta ^{-s}$ and $F(\ell )$ is $\delta ^{s}$-separated.

As a lemma we need the following fact about (generalised) Kakeya sets:

#### Lemma 3.38

Let $t \in [0,1]$, and let $\mathcal {L} \subset \mathcal {A}(2,1)$ be a $(\delta ,1)$-set such that $\operatorname {diam}(\ell \cap B(1)) \sim 1$ for all $\ell \in \mathcal {L}$. Then, the set $U := B(1) \cap \bigcup _{\ell \in \mathcal {L}} [\ell ]_{\delta }$ satisfies $\textrm{Leb}(U) \gtrapprox \delta |\mathcal {L}|$.

#### Proof

For every $\ell \in \mathcal {L}$, let $F(\ell ) \subset \ell \cap B(1)$ be a maximal $\delta$-separated set (in particular $\mathcal {F}(\ell )$ is a $(\delta ,1)$-set). Let $P \subset U \subset B(1)$ be a maximal $\delta$-separated set. Write $\mathcal {F} := \{F(\ell ) : \ell \in \mathcal {L}\}$. Then, $|\mathcal {I}_{2\delta }(P,\mathcal {F})| \gtrsim \delta ^{-1}|\mathcal {L}|$. Combining this with Proposition 3.33 with $t \leqslant s:= 1$, we obtain

$$\begin{aligned} \delta ^{-1}|\mathcal {L}| \lesssim |\mathcal {I}_{2\delta }(P,\mathcal {L})| \lessapprox \sqrt{\delta ^{-1}|P||\mathcal {L}|} + |P|, \end{aligned}$$

which can be rearranged to $|P| \gtrapprox \delta ^{-1}|\mathcal {L}|$. Thus $\textrm{Leb}(U) \gtrsim \delta ^{2}|P| \gtrapprox \delta |\mathcal {L}|$. $\square$

#### Remark 3.39

In the previous proof, we could have also used Theorem 3.22.

#### Theorem 3.40

Let $s \in (0,1]$, and let $\mathcal {L} \subset \mathcal {A}(2,1)$ be such that $\operatorname {diam}(B(1) \cap \ell ) \sim 1$ for all $\ell \in \mathcal {L}$. Set $U := B(1) \cap \bigcup _{\ell \in \mathcal {L}} [\ell ]_{\delta }$. Let $F(\ell ) \subset B(1) \cap \ell$ be a $\delta ^{s}$-dense set for all $\ell \in \mathcal {L}$. Then, the set $F:= \bigcup _{\ell \in \mathcal {L}} F(\ell )$ satisfies

$$\begin{aligned} |F|_{\delta } \gtrsim \delta ^{-(3\,s + 1)/2}\textrm{Leb}(U). \end{aligned}$$

In particular, if $\mathcal {L}$ is a $(\delta ,1)$-set with $|\mathcal {L}| \gtrsim \delta ^{-1}$, then $|F|_{\delta } \gtrapprox \delta ^{-(3\,s + 1)/2}$ (by Lemma 3.38).

#### Proof

**Fig. 3:**
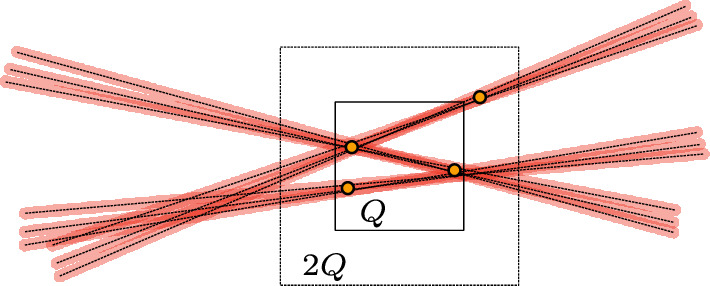
Here $M_{Q} = 3$, even though 9 tubes in total intersect *Q*

Write $\mathcal {T}:= \{[\ell ]_{\delta } \cap B(1): \ell \in \mathcal {L}\}$, so $U:= B(1) \cap \bigcup _{T \in \mathcal {T}} T$. For $Q \in \mathcal {D}_{\delta ^{s}}(U) = \{Q \in \mathcal {D}_{\delta ^{s}}: Q \cap U \ne \varnothing \}$ fixed, we say that two tubes $T,T' \in \mathcal {T}$ with $T \cap Q \ne \varnothing \ne T' \cap Q$ are *Q-distinct* if

$$\begin{aligned} T \cap Q \not \subset [T']_{2\delta }, \end{aligned}$$

see Fig. 3 for intuition. Let $M_{Q}$ be the maximal cardinality of a *Q*-distinct subset of $\mathcal {T}$.

#### Claim 3.41

We have

$$\begin{aligned} M_{Q} \gtrsim \textrm{Leb}(Q \cap U) \cdot \delta ^{-s - 1}, \qquad Q \in \mathcal {D}_{\delta ^{s}}(U). \end{aligned}$$

#### Proof

Let $T_{1},\ldots ,T_{M_{Q}} \in \mathcal {T}$ be a maximal *Q*-distinct collection. Then, every tube $T \in \mathcal {T}$ satisfies $T \cap Q \subset [T_{j}]_{2\delta } \cap Q$ for some $1 \leqslant j \leqslant M_{Q}$. In particular, $Q \cap U$ is contained in the union of the sets $[T_{j}]_{2\delta } \cap Q$ with $1 \leqslant j \leqslant M_{Q}$. The Lebesgue measure of each of these sets is $\lesssim \delta ^{s + 1}$, so $\textrm{Leb}(Q \cap U) \lesssim M_{Q}\delta ^{s + 1}$, as claimed. $\square$

The proof of the next claim is a variant of the 2-ends argument in Remark 2.13.

#### Claim 3.42

We have

$$\begin{aligned} |F \cap 2Q|_{\delta } \gtrsim M_{Q}^{1/2}, \qquad Q \in \mathcal {D}_{\delta ^{s}}(U). \end{aligned}$$

#### Proof

Let $F' \subset F \cap 2Q$ be a maximal $\delta$-separated set. Recall that the points in $F(\ell )$ form a $\delta ^{s}$-net in $\ell \cap B(1)$. Therefore, if $T = [\ell ]_{\delta }$, and $T \cap Q \ne \varnothing$, the set $T \cap 2Q$ contains two distinct points $p,q \in F' \cap 2Q$ with separation $|p - q| \geqslant \delta ^{s}$, see Fig. 3.

Therefore, we obtain a map $T \mapsto (F' \cap 2Q)^{2}$ which associates to every tube $T \in \mathcal {T}$ with $T \cap Q \ne \varnothing$ a pair $(p,q) \in (F' \cap 2Q)^{2}$ with separation $|p - q| \geqslant \delta ^{s}$. It now remains to observe that this map is *C*-to-1 on every collection of *Q*-distinct tubes. Therefore $|F' \cap 2Q|^{2} \gtrsim M_{Q}$, as claimed. $\square$

Finally, observe that $\textrm{Leb}(Q \cap U)^{1/2} \geqslant \delta ^{-s}\textrm{Leb}(Q \cap U)$ for $Q \in \mathcal {D}_{\delta ^{s}}(U)$, so

$$\begin{aligned} |F|_{\delta }&\gtrsim \sum _{Q \in \mathcal {D}_{\delta ^{s}}(U)} |F \cap 2Q|_{\delta } \gtrsim \sum _{Q \in \mathcal {D}_{\delta ^{s}}(U)} M_{Q}^{1/2}\\&\gtrsim \delta ^{-\frac{s + 1}{2}} \sum _{Q \in \mathcal {D}_{\delta ^{s}}(U)} \textrm{Leb}(Q \cap U)^{1/2}\\&\geqslant \delta ^{-\frac{3s + 1}{2}} \sum _{Q \in \mathcal {D}_{\delta ^{s}}(U)} \textrm{Leb}(Q \cap U) = \delta ^{-\frac{3s + 1}{2}} \textrm{Leb}(U). \end{aligned}$$

This completes the proof. $\square$

## Applications and connections of Furstenberg sets

In 2000, Wolff [62, 63] had recently posed the (*s*, 1)-Furstenberg set problem, and it was, e.g. known that every $(\tfrac{1}{2},1)$-Furstenberg set $F \subset \mathbb {R}^{2}$ has $\dim _{\textrm{H}}F \geqslant 1$. The conjecture (now a theorem) states that $\dim _{\textrm{H}}F \geqslant 4/3$. In their influential 2001 paper, Katz and Tao [34] showed that an $\epsilon$-improvement to the bound $\dim _{\textrm{H}}F \geqslant 1$ is logically equivalent (at a $\delta$-discretised level) to an $\epsilon$-improvement in a version of *Falconer’s distance set conjecture*, and also an $\epsilon$-improvement in the $\delta$-discretised version of the *Erdős-Szemerédi sum-product problem*. All of these equivalent $\epsilon$-improvements were shortly afterwards obtained by Bourgain [4].

During the past 20 years, more connections have been discovered, for example to *orthogonal* and *radial projections*. The connection to orthogonal projections is straightforward: Non-trivial results on the $\delta$-discretised Furstenberg set problem formulated in Proposition 3.37 imply non-trivial bounds on the dimension of *exceptional sets of orthogonal projections*. The mechanism is explained in [48, Sect. 3.2]. Notably, the full solution, due to Ren and Wang [53], implies the following sharp bound stated in [53, Theorem 1.2]:

### Theorem 4.1

Let $K \subset \mathbb {R}^{2}$ be analytic. Then, writing $\pi _{e}(x) := x \cdot e$ for $e \in S^{1}$ and $x \in \mathbb {R}^{2}$,

$$\begin{aligned} \dim _{\textrm{H}}\{e \in S^{1} : \dim _{\textrm{H}}\pi _{e}(K) \!<\! \sigma \} \!\leqslant \!\min \{2\sigma \!-\! \dim _{\textrm{H}}K,0\}, \qquad 0 \!\leqslant \!\sigma \!\leqslant \! \min \{\dim _{\textrm{H}}K,1\}. \end{aligned}$$

A weaker estimate was earlier obtained by Kaufman [35].

The following sections contain brief accounts on the implications of Furstenberg set estimates to the (continuous) sum-product problem, to radial projections, and to arithmetic sums of fractal sets on the parabola.

### The sum-product problem

The Erdős-Szemerédi sum-product conjecture [7] asks to prove that if $A \subset \mathbb {Z}$ (original formulation) or $A \subset \mathbb {R}$ (plausible extension) is a finite set, then $\max \{|A + A|,|A \cdot A|\} \gtrsim _{\epsilon } |A|^{2 - \epsilon }$ for all $\epsilon > 0$. This problem remains open and very actively studied. Elekes in the late 90 s [16] connected the problem to the Szemerédi-Trotter incidence bound and allowed him to establish the partial result $\max \{|A + A|,A \cdot A|\} \gtrsim |A|^{5/4}$. This is nowadays far below the state-of-the-art in the discrete version of Erdős and Szemerédi’s problem (see [54]), but his argument has the benefit of extending easily to a “continuum” setting. The main idea is the following observation:

#### Lemma 4.2

Let $A,B,C \subset \mathbb {R}$ be sets. Let $F = (A + B) \times (A \cdot C)$. Then, the family of lines

$$ \mathcal {L}(B,C) := \big \{\ell _{b,c} = \{(x,cx - bc) : x \in \mathbb {R}\}: b\in B, c\in C \big \} $$

has the property that $F\cap \ell _{b,c}$ contains the affine copy of *A* given by $\{(a+b, a\cdot c):a\in A\}$.

#### Proof

Note that $(a + b,a \cdot c) \in \ell _{b,c}$ for all $a,b,c \in \mathbb {R}$, since $c(a + b) - bc = a \cdot c$. Therefore $\{(a + b,a \cdot c) : a \in A\} \subset F \cap \ell _{b,c}$ for all $(b,c) \in B \times C$. $\square$

To use Lemma 4.2, one checks that the line set $\mathcal {L}(B,C) \subset \mathcal {A}(2,1)$ is diffeomorphic to $B \times C$, and in particular $\dim _{\textrm{H}}\mathcal {L}(B,C) = \dim _{\textrm{H}}(B \times C)$. Therefore, Lemma 4.2 tells us that $F = (A + B) \times (A \cdot C)$ is an (*s*, *t*)-Furstenberg set with

$$\begin{aligned} s = \dim _{\textrm{H}}A \quad {\text {and}} \quad t = \dim _{\textrm{H}}(B \times C). \end{aligned}$$

One can now deduce various inequalities about $\dim _{\textrm{H}}F$ using the Furstenberg set theorem. We only consider the case $A = B = C$. Write $s = \dim _{\textrm{H}}A$, and note that $\dim _{\textrm{H}}(A \times A) \geqslant 2s$. Thus, $F = (A + A) \times (A \cdot A)$ is an $(s,2\,s)$-Furstenberg set, and

$$\begin{aligned} \dim _{\textrm{H}}((A + A) \times (A \cdot A)) = \dim _{\textrm{H}}F {\mathop {\geqslant }\limits ^{(3.1)}} \min \left\{ \tfrac{5\,s}{2},s + 1 \right\} . \end{aligned}$$

Since $\dim _{\textrm{H}}(A \times B) \leqslant \dim _{\textrm{H}}A + \dim _{\textrm{P}} B$ (where $\dim _{\textrm{P}}$ is the *packing dimension*), it follows that either

$$\begin{aligned} \dim _{\textrm{H}}(A + A) \geqslant \min \left\{ \tfrac{5\,s}{4},\tfrac{s + 1}{2}\right\} \quad {\text {or}} \quad \dim _{\textrm{P}} (A \cdot A) \geqslant \min \left\{ \tfrac{5\,s}{4},\tfrac{s + 1}{2}\right\} . \end{aligned}$$

Here “$\tfrac{5}{4}$” is Elekes’ exponent. This argument also works well at a $\delta$-discretised level, but we leave the details to the reader (or see [49, Corollary 6.6]). For further recent work on the continuum and $\delta$-discretised sum-product problems, see [39, 43].

### Radial projections and the dimension of quotients

In 2020, the best general lower bounds for the dimension of (*s*, *t*)-Furstenberg sets were the following:

4.3 $$\begin{aligned} \dim _{\textrm{H}}F \geqslant \max \{2s,s + \tfrac{t}{2}\}, \qquad s \in (0,1], \, t \in [0,2]. \end{aligned}$$

The 2*s*-bound is a special case of Corollary 3.35, whereas the $(s + \tfrac{t}{2})$-bound is due to Héra [29] (see also [41] for a partial result). In 2021, Shmerkin and myself [48] proved the following “$\epsilon$-improvement” over the 2*s*-bound:

#### Theorem 4.4

For $s \in (0,1)$ and $t \in (s,2]$, the Hausdorff dimension of every (*s*, *t*)-Furstenberg set $F \subset \mathbb {R}^{2}$ satisfies $\dim _{\textrm{H}}F \geqslant 2s + \epsilon (s,t)$ for some $\epsilon (s,t) > 0$.

Theorem 4.4 was applied in [50] to prove a new *radial projection theorem*, which, in turn, played a role in the solution of the full Furstenberg set problem (see [48, 53]). I will now explain (very informally) the connection between Theorem 4.4 and radial projections.

For $x \in \mathbb {R}^{2}$, the *radial projection to x* is the map $\pi _{x} :\mathbb {R}^{2} {\setminus } \{x\} \rightarrow S^{1}$ defined by $\pi _{x}(y) = (x - y)/|x - y|$. A good way to think about radial projections is the following: if $F \subset \mathbb {R}^{2}$ and $x \in \mathbb {R}^{2} {\setminus } F$, then $\dim _{\textrm{H}}\pi _{x}(F) = \dim _{\textrm{H}}\mathcal {L}_{x}$, where

$$\begin{aligned} \mathcal {L}_{x} = \{\ell \in \mathcal {A}(2,1) : x \in \ell {\text { and }} F \cap \ell \ne \varnothing \}. \end{aligned}$$

In [50], we proved the following:

#### Theorem 4.5

Let $E,F \subset \mathbb {R}^{2}$ be disjoint Borel sets such that *E* is not contained on any line. Then, $\sup _{x \in E} \pi _{x}(F) = \min \{\dim _{\textrm{H}}E,\dim _{\textrm{H}}F,1\}$.

The following argument is a very rough indication of how the $\epsilon$-improvement in the Furstenberg set problem, Theorem 4.4, appears in the proof of Theorem 4.5.

#### “Proof” of Theorem 4.5

Write $s:= \min \{\dim _{\textrm{H}}E,\dim _{\textrm{H}}F,1\}$. If necessary replacing *F* by an *s*-dimensional subset, we may assume that $\dim _{\textrm{H}}F = s$. To reach a contradiction, assume that $\dim _{\textrm{H}}\pi _{x}(F) = s - \eta$ for all $x \in E$, where $\eta > 0$. In other words $\dim _{\textrm{H}}\mathcal {L}_{x} = s - \eta$ for all $x \in E$.

The set $\mathcal {L} = \bigcup _{x \in E} \mathcal {L}_{x} \subset \mathcal {A}(2,1)$ is a “dual” $(s - \eta ,s)$-Furstenberg set: It contains an $(s - \eta )$-dimensional line family incident to every point in the *s*-dimensional set *E*. Since $s - \eta < s$, Theorem 4.4 implies

$$\begin{aligned} \dim _{\textrm{H}}\mathcal {L} \geqslant 2(s - \eta ) + \epsilon \end{aligned}$$

for some $\epsilon := \epsilon (s - \eta ,s) > 0$. Or does it? A potential problem is that $\epsilon (0,s) = 0$ in Theorem 4.4, so if $\eta = s$ (i.e. $\dim _{\textrm{H}}\pi _{x}(F) \equiv 0$ for $x \in E$), we are in trouble. However, we may assume here that $\eta \leqslant \tfrac{s}{2}$. This part of the argument uses the hypothesis that *E* is not contained on a line, and is based on a much earlier result [44].

Now we pose a rather unrealistic simplifying assumption: since $\dim _{\textrm{H}}\mathcal {L}_{x} = s - \eta$, but $\dim _{\textrm{H}}F = s$, it is somewhat reasonable to expect that $\dim _{\textrm{H}}(F \cap \ell ) \geqslant \eta$ for $\ell \in \mathcal {L}_{x}$. If this were the case, as we now assume, then *F* is an $(\eta ,t)$-Furstenberg set with $t = \dim _{\textrm{H}}\mathcal {L} \geqslant 2(s - \eta ) + \epsilon$. Consequently, by the second “classical” bound in (4.3),

$$\begin{aligned} s = \dim _{\textrm{H}}F \geqslant \eta + \frac{2(s - \eta ) + \epsilon }{2} = s + \frac{\epsilon }{2}. \end{aligned}$$

This contradiction completes the “proof” of Theorem 4.5. $\square$

As a corollary, we obtain the following sum-quotient estimate:

#### Corollary 4.6

Let $A, B\subset \mathbb {R}$ be Borel sets. Then

$$ \dim _{\textrm{H}}\left( \frac{A-B}{A-B}\right) \geqslant \min \{\dim _{\textrm{H}}A + \dim _{\textrm{H}}B, 1\}. $$

#### Proof

We may assume that both *A*, *B* contain at least two points. We apply Theorem 4.5 to the sets $E = -A \times B$ and $F = -B \times A$. Since *E* is a Borel set not contained on a line, for every $\epsilon > 0$, there exists a point $x = (-a,b) \in E$ such that

$$\begin{aligned} \dim _{\textrm{H}}\pi _{x}(F {\setminus } \{x\}) \geqslant \min \{\dim _{\textrm{H}}E,\dim _{\textrm{H}}F,1\} - \epsilon \geqslant \min \{\dim _{\textrm{H}}A + \dim _{\textrm{H}}B,1\} - \epsilon . \end{aligned}$$

Now, it remains to observe that $\dim _{\textrm{H}}\pi _{x}(F {\setminus } \{x\})$ agrees with the dimension of “slopes” spanned between the point $x = (-a,b)$ and the set $F {\setminus } \{x\}$, namely

$$\begin{aligned} \left\{ \tfrac{a' \!-\! b}{-\!b' \!-\! (-a)} : (-b',a')\! \in \! (-B \times A) {\setminus } \{(-a,b)\} \right\} \!=\! \left\{ \tfrac{a' - b}{a - b'} : (-b',a') \in (-B \times A) {\setminus } \{(-a,b)\} \right\} . \end{aligned}$$

Since the quotient set $(A - B)/(A - B)$ contains all such slopes, the corollary follows. $\square$

### Arithmetic sums of subsets of the parabola

The Furstenberg set problem has some hidden “curvature”, which can be brought to light by the following observation. The map $\Psi (x,y):= (x,y^{2} - x)$ sends every non-vertical line $\ell \subset \mathbb {R}^{2}$ to some translate of the standard up-ward pointing parabola $\mathbb {P} = \{(x,x^{2}): x \in \mathbb {R}\}$. More precisely, if $\ell _{(x_{0},y_{0})}:= \{(x,y): y = y_{0} + 2x_{0}(x - x_{0})\}$ is the line with slope $2x_{0}$ passing through $(x_{0},y_{0})$, then

$$\begin{aligned} \Psi (\ell _{(x_{0},y_{0})}) = \Psi (x_{0},y_{0}) + \mathbb {P}. \end{aligned}$$

The map $\Psi$ is locally bi-Lipschitz, so *s*-dimensional subsets of lines are carried to *s*-dimensional subsets of the corresponding parabolas (for an alternative argument, observe that $\Psi$ leaves the first coordinate fixed). The map $\Psi$ also sends every *t*-dimensional set of lines to a family of parabolas of the form $\{x + \mathbb {P}\}_{x \in X}$, where $\dim _{\textrm{H}}X = t$.

Using the map $\Psi$, and its properties described above, one obtains (with the argument of [47, Sect. 1.1]) the following corollary of the Furstenberg set theorem:

#### Corollary 4.7

Let $F \subset \mathbb {P}$ be a set with $\dim _{\textrm{H}}F = s \in (0,1]$, and let $E \subset \mathbb {R}^{2}$ be a set with $\dim _{\textrm{H}}E = t \in [0,2]$. Then,

4.8 $$\begin{aligned} \dim _{\textrm{H}}(E + F) \geqslant \min \left\{ s + t,\tfrac{3s + t}{2},s + 1 \right\} . \end{aligned}$$

In particular, $$\dim _{\textrm{H}}(F + F + F) \geqslant \min \{\tfrac{5s}{2},s + 1\}$$.

#### Proof

The set $\Psi ^{-1}(E + F)$ is an (*s*, *t*)-Furstenberg set, so (4.8) is implied by the Furstenberg set theorem. The bound $\dim _{\textrm{H}}(F + F + F) \geqslant \min \{\tfrac{5s}{2},s + 1\}$ follows by applying (4.8) to $E = F + F$ and *F* (and noting first that $\dim _{\textrm{H}}E \geqslant 2s$). $\square$

#### Remark 4.9

The bound $\dim _{\textrm{H}}(F + F + F) \geqslant \min \{\tfrac{5s}{2},s + 1\}$ is a continuum analogue of an observation of Bourgain and Demeter [5, Proposition 2.15]. They showed that if $\Lambda \subset \mathbb {P}$ is a finite set, then $|\Lambda + \Lambda + \Lambda | \gtrsim |\Lambda |^{5/2}$. They also ask in [5, Question 2.13] whether $|\Lambda + \Lambda + \Lambda | \gtrsim _{\epsilon } |\Lambda |^{3 - \epsilon }$, and deduce a positive answer from the $\ell ^{2}$-decoupling theorem if $\Lambda$ is $|\Lambda |^{-C}$-separated. Analogously, it seems unlikely that the bound for $\dim _{\textrm{H}}(F + F + F)$ in Corollary 4.7 is sharp. For further literature on this topic, see [10, 12, 47].

## Data Availability

No datasets were generated or analysed during this study.

## Footnotes

1 If $[p,q] \in E(\ell _{1}) \cap E(\ell _{2})$, then the segment [*p*, *q*] is contained in $[\ell _{2}]_{\delta } \cap [\ell _{2}]_{\delta }$, which forces $\angle (\ell _{1},\ell _{2}) \lesssim \sqrt{\delta }$.

2 If *Z* contained more lines than its degree, then almost every lines of $\mathbb {R}^{2}$ would be contained in *Z*. This would lead to $Z = \mathbb {R}^{2}$, and therefore $\textrm{poly} \equiv 0$ contrary to Lemma 2.11.

3 First find a sequence $\{Q_{j}\}$ of arbitrary dyadic cubes covering *K* with $\sum \ell (Q_{j})^{s} \leqslant \tfrac{1}{2}2^{-k_{0}s}$. Enlarge the cubes slightly to make them open, but still retaining $\sum \ell (Q_{j})^{s} \leqslant 2^{-k_{0}s}$. Finally, pick a finite sub-cover, and let $\delta > 0$ be the smallest side-length in that sub-cover.
